# Concept annotation in the CRAFT corpus

**DOI:** 10.1186/1471-2105-13-161

**Published:** 2012-07-09

**Authors:** Michael Bada, Miriam Eckert, Donald Evans, Kristin Garcia, Krista Shipley, Dmitry Sitnikov, William A Baumgartner, K Bretonnel Cohen, Karin Verspoor, Judith A Blake, Lawrence E Hunter

**Affiliations:** 1 Department of Pharmacology, University of Colorado Anschutz Medical Campus, Aurora, CO, USA; 2 Department of Linguistics, University of Colorado Boulder, Boulder, CO, USA; 3 Mouse Genome Informatics, The Jackson Laboratory, Bar Harbor, ME, USA; 4 Victoria Research Lab, National ICT Australia, Melbourne, VIC, 3010, Australia

## Abstract

**Background:**

Manually annotated corpora are critical for the training and evaluation of automated methods to identify concepts in biomedical text.

**Results:**

This paper presents the concept annotations of the Colorado Richly Annotated Full-Text (CRAFT) Corpus, a collection of 97 full-length, open-access biomedical journal articles that have been annotated both semantically and syntactically to serve as a research resource for the biomedical natural-language-processing (NLP) community. CRAFT identifies all mentions of nearly all concepts from nine prominent biomedical ontologies and terminologies: the Cell Type Ontology, the Chemical Entities of Biological Interest ontology, the NCBI Taxonomy, the Protein Ontology, the Sequence Ontology, the entries of the Entrez Gene database, and the three subontologies of the Gene Ontology. The first public release includes the annotations for 67 of the 97 articles, reserving two sets of 15 articles for future text-mining competitions (after which these too will be released). Concept annotations were created based on a single set of guidelines, which has enabled us to achieve consistently high interannotator agreement.

**Conclusions:**

As the initial 67-article release contains more than 560,000 tokens (and the full set more than 790,000 tokens), our corpus is among the largest gold-standard annotated biomedical corpora. Unlike most others, the journal articles that comprise the corpus are drawn from diverse biomedical disciplines and are marked up in their entirety. Additionally, with a concept-annotation count of nearly 100,000 in the 67-article subset (and more than 140,000 in the full collection), the scale of conceptual markup is also among the largest of comparable corpora. The concept annotations of the CRAFT Corpus have the potential to significantly advance biomedical text mining by providing a high-quality gold standard for NLP systems. The corpus, annotation guidelines, and other associated resources are freely available at http://bionlp-corpora.sourceforge.net/CRAFT/index.shtml.

## Background

With the digitalization of much of the biomedical literature, automated processing of journal publications has become increasingly important in biomedical research. Biomedical researchers struggle to keep abreast of the exponentially growing literature, due to not only its sheer scale but also to the expanding range of disciplines and journals relevant to a typical research question. Biomedical publications, like most texts, are fraught with synonymy, polysemy, ambiguity, and complexity. Transformation of these texts into formal representations of the contained knowledge makes possible the application of sophisticated computational methods that assist researchers and advance science. Substantial progress in biomedical natural-language processing (NLP), particularly in the tasks of information retrieval, concept recognition, and information extraction [1-5] raises the possibility of creating formal representations for the entire biomedical literature.

Development of formal ontologies for the representation of domain-specific knowledge has also made substantial progress [6]. Among the most ambitious of these efforts are the Open Biomedical Ontologies (OBOs), a set of ontologies whose domains include anatomy, biological processes and functions, cells and cellular components, chemicals, phenotypes and diseases, and experiments and procedures. These ontologies are largely constructed in a community-driven approach, and their developers commit to a common set of attributes including openness, shared syntax, clear versioning, demarcated content, and clear definition [7]. Millions of genes, gene products, and biomedical data sets have been annotated with ontological terms, and these annotations are widely used as the basis for high-throughput data analysis. In particular, calculations of enrichment of Gene Ontology (GO) terms in sets of differentially expressed genes are widely used [8-10], and more sophisticated uses of formal knowledge representations in data analysis are beginning to be published (*e.g.*, [11]).

Manually annotated, or “gold-standard”, corpora are increasingly important for the development of sophisticated NLP systems, both as training data and for evaluative purposes. Use of manually annotated biomedical corpora in NLP research has consistently led to improved results. In a study by Tomanek *et al.*, the accuracy of tokenization of a test set of biomedical text increased from 71.5% when their tool was trained on a corpus that was tokenized using newspaper language patterns to 95.9% when their tool was trained on a corpus whose tokenization was biomedically motivated [12]. Kulick *et al.* showed that accuracy of part-of-speech annotation of biomedical text increased from 88.53% to 97.33% on test abstracts when their tagger was retrained after the training corpus was manually checked and corrected [13], and Coden *et al.* found that adding a small biomedical annotated corpus to a large general-English one increased accuracy of part-of-speech tagging of biomedical text from 87% to 92% [14]. Lease and Charniak demonstrated large reductions in unknown word rates and large increases in accuracy of part-of-speech tagging and parsing when their systems were trained with a biomedical corpus as compared to only general-English and/or business texts [15]. It was shown by Roberts *et al.* that the best results in recognition of clinical concepts (*e.g.*, conditions, drugs, devices, interventions) in biomedical text, ranging from 10% below to 11% above the interannotator-agreement scores for the gold-standard test set, were obtained with the inclusion of statistical models trained on a manually annotated corpus as compared to dictionary-based concept recognition solely [16]. Craven and Kumlein found generally higher levels of precision of extracted biomedical assertions (*e.g.*, protein-disease associations and subcellular, cell-type, and tissue localizations of proteins) for Naïve-Bayes-model-based systems trained on a corpus of abstracts in which such assertions were manually annotated, as compared to a basic sentence-cooccurrence-based method [17].

In recognition of the importance of such corpora, the Colorado Richly Annotated Full-Text (CRAFT) Corpus, a collection of 97 full-length, open-access biomedical journal articles selected from the regular annotation stream of a major bioinformatics resource, has been manually annotated to indicate references to concepts from multiple ontologies and terminologies. Specifically, it contains annotations indicating all mentions in each full-length article of the concepts from nine prominent ontologies and terminologies: the Cell Type Ontology (CL, representing cells) [18,19], the Chemical Entities of Biological Interest ontology (ChEBI, representing chemicals, chemical groups, atoms, subatomic particles, and biochemical roles and applications) [20], the NCBI Taxonomy (NCBITaxon, representing biological taxa) [21], the Protein Ontology (PRO, representing proteins and protein complexes), the Sequence Ontology (SO, representing biomacromolecular sequences and their associated attributes and operations) [22,23], the entries of the Entrez Gene database (EG, representing genes and other DNA sequences at the species level) [24], and the three subontologies of the GO, *i.e.*, those representing biological processes (BP), molecular functions (MF), and cellular components (CC) [25,26].

The first public release of the CRAFT Corpus includes the annotations for 67 of the 97 articles, reserving two sets of 15 articles for future text-mining competitions (after which these too will be released)., This corpus is among the largest gold-standard annotated biomedical corpora, and unlike most others, the journal articles that comprise the documents of the corpus are marked up in their entirety and range over a wide range of disciplines, including genetics, biochemistry and molecular biology, cell biology, developmental biology, and even computational biology. The scale of conceptual markup is also among the largest of comparable corpora. While most other annotated corpora use small annotation schemas, typically comprised of a few to several dozen classes, all of the conceptual markup in the CRAFT Corpus relies on large ontologies and terminologies nearly in their entirety, creating an unprecedentedly rich semantic resource. Since we have been guided by marking up textual mentions with their directly corresponding ontological and terminological concepts, these mentions are marked up without loss of knowledge.. All of the concept annotations of all terminologies used were created using a single set of guidelines, making clear which spans of text are to be marked up and what the span boundaries should be, which has resulted in high interannotator agreement. Along with the syntactic [27] and coreferential [28] annotations that have been created for the same set of journal articles, the concept annotations of the CRAFT Corpus have the potential to significantly advance biomedical text mining by providing a high-quality gold standard for bioNLP systems.

Following this brief introduction, we will present the salient statistics for the conceptual markup of the corpus in the form of counts of concept annotations and of unique annotated concepts for each of the vocabularies used, as well as the formats in which this markup is being released. This is followed by an in-depth comparison of the concept annotations of the CRAFT Corpus to those of other publicly available manually annotated gold-standard biomedical corpora as well as several other relevant projects, along with a discussion of the aspects of our concept annotations that we claim are prominent factors in their being a significant contribution to the bioNLP community. Ongoing and future work is then briefly described, followed by our conclusions. The primary text of our paper ends with methodology with regard to corpus assembly, terminology selection, creation of annotation guidelines, and creation of the conceptual markup. Finally, as supplementary material, we provide an extensive presentation of our concept-annotation guidelines and a spreadsheet of our interannotator-agreement statistics in detail.

## Results

The articles in the CRAFT Corpus have been completely marked up with the full sets of concepts^a^ (minus a small number of terms) of nine biomedical ontologies and terminologies. Here we present annotation-count and interannotator-agreement statistics for the conceptual annotation. In a companion paper [27] we present the syntactic annotation (*i.e.*, of sentences, tokens and parse trees) of the CRAFT Corpus and studies in which it was used to train high-performing models, providing indirect evidence of its high quality.

### Concept annotation statistics

Table 1 presents statistics for the counts of concept annotations in total and also for each ontology and terminology in the 67 articles constituting the initial public release of the CRAFT Corpus. These data show that the mentions of the concepts of these ontologies and terminologies are abundant: There is a total of 99,907 concept annotations in these articles, ranging from 4,062 annotations of GO MF concepts to 22,090 annotations of SO concepts. Furthermore, as the initial public release consists of approximately two thirds of the articles in the entire corpus, the annotations in the entire corpus total more than 140,000 (not shown). There is an average of 1,491 annotations of the concepts from all of these terminologies per article, ranging from an average of 61 mentions of GO MF concepts per article to 330 mentions of SO concepts per article. However, as the values of the median counts of annotations per article are lower than their corresponding averages per article, and in most cases substantially so, these averages are skewed upward by smaller numbers of articles with very high annotation counts. The last two columns of Table 1, which present minimum and maximum counts per article, indicate that there is indeed a very wide range of annotations per article across the articles for all of these terminologies.

**Table 1:** Counts of annotations

**terminology**	**# total annotations**	**average # annotations per article**	**median # annotations per article**	**minimum # annotations per article**	**maximum # annotations per article**
ChEBI	8,137	121	94	11	486
CL	5,760	86	58	0	435
Entrez Gene	12,277	183	155	3	543
GO BP^a^	16,184	241	194	14	738
GO CC	8,354/4,707^b^	125/70	97/51	9/0	499/322
GO MF	4,062	61	42	2	403
NCBITaxon^c^	7,449	111	91	12	378
PRO	15,594	233	207	4	704
SO^d^	22,090	330	328	72	935
all	99,907	1,491^e^			

^a^We are still in the process of reviewing and editing the GO BP & MF annotations for the official 1.0 version release; therefore, the statistics for these will likely change. We will update annotation statistics on the project Web site as needed.

^b^We have calculated statistics for the GO CC project both with and without the annotations of cell (GO:0005623), as these account for over half of the annotations of this project. In addition to skewing these statistics, since this is such a trivial concept that is also being annotated in the CL project, users may wish to exclude these annotations for training and evaluation of systems.

^c^In addition to the hundreds of thousands of organism entries, the NCBI Taxonomy also has a small taxonomy of types of biological taxa (*e.g.*, phylum, genus, subgenus). For the NCBI Taxonomy pass, there are also a small number of annotations of the mentions of these taxonomic concepts in the articles; however, we have excluded these in these statistics.

^d^For the SO statistics, the independent_continuant annotations (as described in the Methodology) were excluded from the analysis.

^e^The averages of the total number of annotations per article and of unique concepts per article were calculated simply by adding up the averages for each terminological annotation pass.

Counts of annotations and of average, median, minimum, and maximum counts of annotations per article for the 67 articles constituting the initial public release of the CRAFT Corpus.

Table 2 presents statistics for the counts of unique concepts mentioned in these articles, both totaled and for each ontology and terminology in these 67 articles. These data show that these concept mentions are also diverse: There is a total of 4,319 unique concepts from these ontologies and terminologies mentioned in these articles, ranging from 149 unique NCBI Taxonomy concepts to 1,024 unique Entrez Gene concepts. There is an average of 192 unique concepts of these ontologies and terminologies mentioned per article, ranging from 7 unique CL concepts per article to 41 unique SO concepts per article. As with the annotation counts, there is a wide range of unique concepts mentioned per article across the articles for all of these terminologies, as indicated by their minimum and maximum counts of unique concepts mentioned per article. However, the median counts of unique concepts mentioned per article overall are very close to their corresponding average values, indicating that the averages are not skewed much by outlier values.

**Table 2:** Counts of unique annotated concepts

**terminology**	**# total unique concepts**	**average # unique concepts per article**	**median # unique concepts per article**	**minimum # unique concepts per article**	**maximum # unique concepts per article**
ChEBI	553	32	28	4	90
CL	155	7	6	0	22
Entrez Gene	1,024	18	17	1	95
GO BP	758	40	40	11	91
GO CC	213/212	12/11	10/9	1/0	33/32
GO MF	318	13	12	1	37
NCBITaxon	149	11	10	3	49
PRO	889	18	19	1	44
SO	260	41	43	8	89
all	4,319	192			

Counts of unique mentioned concepts and of average, median, minimum, and maximum counts of unique mentioned concepts per article for the 67 articles constituting the initial public release of the CRAFT Corpus.

### Interannotator-agreement statistics

**Figure 1:**
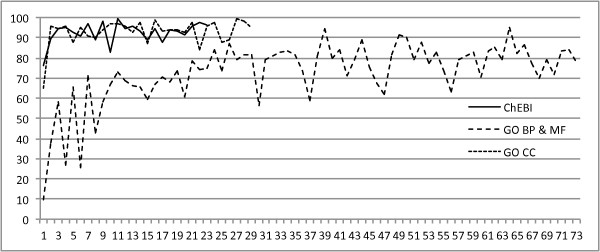
**IAA statistics for ChEBI and GO BP/MF, and GO CC markup.** Plot of IAA versus number of training sessions/meetings (approximately weekly) for annotation of the corpus with the ChEBI ontology, GO BP & MF, and CC. IAA has been calculated as F-score, which is the harmonic mean of precision and recall.

**Figure 2:**
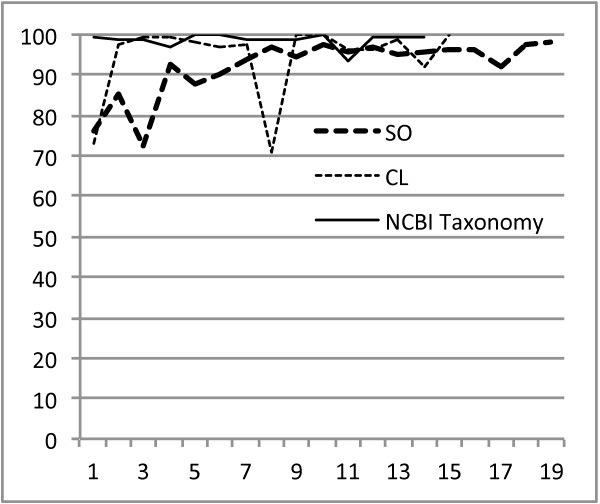
**IAA statistics for CL, NCBITaxon, and SO markup.** Plot of IAA versus number of training sessions/meetings (approximately weekly) for annotation of the corpus with the SO, CL, and NCBI Taxonomy. IAA has been calculated as F-score, which is the harmonic mean of precision and recall.

Figures 1 and 2 illustrate that the use of our concept annotation guidelines (which we present in detail as supplementary material) has enabled consistently high interannotator agreement after a short initial period of working with a newly encountered ontology. Our annotators, who are domain experts, not knowledge engineers (nor linguists), were able to quickly reach and with occasional exception remain at a 90 + % IAA level for all of the terminological annotation passes except for the challenging GO BP & MF pass^b^. Oscillations in these figures are partly explained by the fact that an annotator may make the same type of error many times in a given article, which can strongly affect IAA statistics. For example, a given article often has many mentions of some concept, and two annotators might consistently annotate these mentions differently, leading to a considerable drop in IAA. For example, the large drop seen in the eighth data point for the CL project is almost wholly attributable to the consistently discrepant annotation of the several dozen mentions of polymorphonuclear leukocytes/PMNs in one article. (One annotator marked up these mentions using CL:granulocyte (CL:0000094) and the other with CL:mature neutrophil (CL:0000096), one of its subclasses.) In addition to Figures 1 and 2 within this paper, we have included a spreadsheet of the precise IAA statistics for all of the annotation passes as supplementary material (Additional file 1: Doc1).

This degree of IAA is impressive, given that the annotation schemas (*i.e.*, the contents of the target ontologies) are very large (ranging from ~800 to hundreds of thousands of concepts) as compared to the typical textual annotation project, which uses a schema of no more than dozens of classes. Furthermore, a very strict standard of matching was used in the calculation of these IAA statistics: A given pair of annotations was considered a match only if they used the exact same class/term and specified the exact same text span. For many of the mismatches (which result in the lowering of IAA), the given pair of annotations used closely related classes (*e.g.*, a class and its subclass) and/or had only slightly different text spans; thus, even a slight relaxing of the matching criteria would result in even higher IAA figures.

As presented in the Methodology section, most of these data points are single-blind statistics, in which the lead semantic annotator inspected the markup of the annotators, edited (by adding, deleting, or modifying) markup with which he disagreed, and calculated the agreement between the original markup and the edited version. We have also annotated a small number of articles in a double-blind fashion, including the last three articles of the corpus (corresponding to the last three data points of Figure 1) annotated with the BP and MF branches of the GO, which resulted in IAAs of 83.4%, 83.9%, and 78.0%, in concordance with previous data points, as can be seen in this figure. These (albeit limited) data suggest that the single-blind IAAs are unlikely to be biased by a considerable amount.

A primary reason we have not performed double-blind annotation for the majority of the articles of the corpus is simply a lack of resources; as it is, the creation of this semantic markup has entailed tens of thousands of person-hours over more than four years of effort. Additionally, for the creation of a gold-standard annotated corpus, we have found it crucial for a knowledge engineer to thoroughly review the markup using the concepts of these ontologies. It is necessary not only to have a broad familiarity with the ontologies being used but also to have a deeper understanding of their representational subtleties and assumptions that domain experts may very well never even consider much less make sense of by themselves. For example, the ChEBI ontology contains a subgraph for amino acids as discrete molecules (*e.g.*, CHEBI:serine), another for those as amino-acid residues within peptide chains (*e.g.*, CHEBI:serine residue), and another for the molecules' derived substituent groups (*e.g.*, CHEBI:seryl group), and using these correctly and consistently at first may not be at all obvious; indeed, the annotator using ChEBI to mark up the articles of the CRAFT Corpus incorrectly used the molecular terms for many of the residue mentions (*e.g.*, as in "serine 132") before the semantic lead observed this and provided guidance. Additionally, many ontologies have their own idiosyncrasies and often even ambiguous or dubious modeling, and expertise in knowledge engineering is very valuable in deciding how to deal with these representational issues with regard to their use in text annotation [31,32]. The CRAFT Corpus was constructed on the assumption that a consensus set of annotations resulting from single-blind evaluation by a competent knowledge engineer of ontology-based markup created by a domain expert would result in a higher-quality annotated corpus than a consensus set of annotations resulting from double-blind annotation by multiple domain experts. The soundness of this approach is supported by recent studies by Dligach *et al.* that have shown that single-annotating more data as opposed to double annotation is more cost-effective in improving system performance [33] and that double annotation can be greatly reduced without loss of system performance [34].

### Distribution formats

The CRAFT Corpus concept annotations have been released in multiple formats to promote ease of use and community uptake. The release includes both the full-text documents and sets of conceptual and syntactic annotation artifacts accompanying each document. All CRAFT Corpus documents are drawn from the PubMed Central (PMC) Open Access subset [35], licensed to allow redistribution. The CRAFT Corpus release includes two versions of each document: an XML version provided by PMC and a more human-readable plain-text version derived from the XML, the latter of which was used by the annotators to generate concept markup. The character offsets referenced by the annotations are therefore relative to the plain-text documents and not the original XML; inclusion of the XML format in the distribution is for provenance only.

We have provided these concept annotations in both the XML-based GENIA Project Markup Language (GPML) [36], and in the W3C-standard Resource Description Framework (RDF). CRAFT Corpus annotations in GPML will allow quick uptake by users with software based on the well-known GENIA corpus, and the version in RDF will open the corpus up to the Semantic Web community. It is important to note that because discontinuous annotations (*i.e.*, annotations composed of two or more disconnected text spans) cannot be unambiguously represented in GPML, we have excluded all such annotations from this version; thus, the set of concept annotations in the GPML version of the CRAFT Corpus is be regarded as incomplete. An XML-based format produced by the Knowtator annotation tool [37], which is also widely used in the text annotation community, is provided as well.

To facilitate use in NLP generally, we have also released CRAFT tools for the Unstructured Information Management Architecture (UIMA) [38], a popular open-source middleware layer for text processing. The CRAFT Corpus documents and annotations have been bundled as serialized UIMA Common Analysis Structures (CASes), and we provide a UIMA collection-reader component for them.

For browsing and exploratory purposes, we plan to integrate the CRAFT Corpus with two online resources: U-Compare [39] and Massachusetts General Hospital’s Document Metadata Organizer (DOMEO) application, formerly called the SWAN annotation tool [40]. U-Compare is a Web-based NLP system relying on UIMA that allows users to set up and run text-processing pipelines using a variety of tools over a variety of corpora. DOMEO is a Web-enabled annotation tool designed for markup of scientific articles with ontology terms and will allow an easy introduction to the corpus by facilitating browsing of its data. Furthermore, we have already integrated the CRAFT Corpus with BRAT (brat rapid annotation tool) [41], with which our concept annotations can be browsed online at http://compbio.ucdenver.edu/Craft. Note, however, that BRAT cannot render discontinuous annotations (*i.e.*, annotations consisting of two or more disconnected text spans) properly. For each discontinuous annotation, the shortest span of text encompassing all of the text spans is instead highlighted. However, in BRAT, if the cursor hovers over an icon indicating an annotation, a window pops up specifying the annotated text, the concept with which this text is annotated, and the terminology of which this concept is a member; for discontinuous annotations, the correct disconnected spans of text are displayed as the last line in this popup window.

The corpus, annotation guidelines, and other associated resources are freely available at http://bionlp-corpora.sourceforge.net/CRAFT/index.shtml.

## Discussion

CRAFT can be compared to a number of previously released corpora. We focus primarily on biomedical corpora, as these are obviously most directly related: ABGene [42], BioInfer [43], the CLEF Corpus [44], the FetchProt Corpus [45], the Fourth i2b2/VA Challenge corpus [46], GENETAG [47], GENIA [48,49], GREC [50], the ITI TXM PPI and TE corpora [51], MedPost [52], the PennBioIE Oncology and CYP v1.0 corpora [13], and the Yapex corpus [53]. Though it bills itself as a “silver standard”, due to its vast scale we also compare CRAFT to the output of the Collaborative Annotation of a Large Biomedical Corpus (CALBC), an effort at constructing a very large biomedical corpus through “harmonization” of the automatically generated annotations of five systems [54]. Finally, though it focuses on newswire articles, we also compare our corpus to OntoNotes Release 2.0 here, as it is analogously a large-scale manually created corpus project with multiple types of semantic and syntactic annotation [55,56]. Table 1 summarizes some criteria by which we compare CRAFT to other corpora.

Comparison of corpora in terms of total numbers of words/tokens is summarized in Table 3. The full corpus contains ~790,000 tokens, and the initial release contains more than 560,000; they are larger than nearly all gold-standard annotated corpora (for which we could find published numbers), including GENETAG, OntoNotes, GENIA, the PennBioIE Oncology and CYP Corpora, the MedPost Corpus, and BioInfer. The only corpora larger than ours by this criterion is the silver-standard CALBC corpus, with ~16,000,000 tokens, and the gold-standard ITI TXM PPI and TE Corpora, with ~2,000,000 and ~1,900,000 tokens, respectively; however, the counts of the ITI TXM corpora include all versions of the subset of documents that were multiply annotated (independently, for IAA calculation), and, as discussed later, not all sections of the component documents of these corpora were annotated.

**Table 3:** Concept annotation attributes of corpora

**corpus/corpora**	**total # words/tokens**	**# & type of documents**	**domain(s)**	**annotation concept schema(s)**	**total # concept annotations**
CRAFT Corpus (full/initial release)	~790,000/~560,000	97/67 articles	sources of MGI annotations of mouse genes/gene products	Open Biomedical Ontologies (CL, ChEBI, SO, PRO, GO BP/CC/MF, NCBITaxon), Entrez Gene	~140,000/~100,000
ABGene		4,265 sentences		n/a	~8,200
BioInfer	~34,000/~30,000^f^	1,100 sentences	protein-protein interactions	~100 entity classes, ~100 relationships	~6,300 named entities, ~2,700 relationships^g^
CALBC corpus	~16,000,000	150,000 abstracts	immunology	UniProt, NCBITaxon, UMLS^h^	~2,700,000
CLEF Corpus		various^i^	clinical/cancer data	6 concept types	
FetchProt Corpus		200 articles	protein tyrosine kinase activity	10 concept types, UniProt	~3,800
4^th^ i2b2/VA Challenge Corpus		~750 discharge summaries	clinical data	3 concept types	~2,000
GENETAG	~548,000	20,000 sentences		n/a	~25,000 genes/proteins, ~19,000 alternative lexical forms
GENIA 3.0	~440,000	2,000 abstracts	human blood-cell transcription factors	35 entity classes, 34 process classes	~93,000 entities, ~36,000 events
GREC		240 abstracts	*E. coli* gene regulation	433 classes	~5,000
ITI TXM PPI/TE Corpora	~2,000,000/ ~1,900,000	217/238 articles	protein-protein interactions/tissue expression	9/13 concept types, Entrez Gene, RefSeq^j^, ChEBI, MeSH, NCBITaxon^k^	~160,000/~164,000
MedPost	~156,000				
OntoNotes 2.0	~500,000	1,000 newswire documents	English & Chinese news	1000 s of WordNet senses, 50 concept types^l^	~58,000 verbs^m^
PennBioIE Oncology/CYP v1.0 Corpora	~381,000 (~327,000)/~313,000 (~274,000)	1,414/1,100 abstracts	medical genetics of oncology/inhibition of cytochrome P450 enzymes	n/a	
Yapex Corpus		200 abstracts	protein-protein interactions	n/a	~3,700

^f^BioInfer has ~34,000 tokens total, and ~30,000 excluding punctuation.

^g^BioInfer has ~6,300 named-entity annotations and ~2,700 annotations of what are termed relationships but that might more properly be conceptualized as process or state classes and thus are included here, totaling ~9,000 concept annotations.

^h^In the CALBC corpus, NCBI Taxonomy and UMLS concepts were respectively used to mark up species and disease mentions.

^1^The CLEF Corpus is composed of many types of medical documents: 2 entire patient records (themselves composed of 9 narratives, 1 imaging report, 7 histopathology reports, and associated data) and 50 each of clinical narratives, histopathology reports, and imaging reports.

^j^The annotators of the ITI TXM Corpora attempted to assign Entrez Gene IDs to gene annotations and RefSeq IDs to annotations of proteins, mRNAs, and cDNAs (although it is admitted that this assignment was very time-consuming and thus was not performed on the training subset of the PPI Corpus).

^k^The annotators of the ITI TXM Corpora used ChEBI, MeSH, and NCBI Taxonomy concepts for drug, tissue, and sequence mentions.

^l^In OntoNotes, the 700 most frequent polysemous verbs and 1,100 most frequent polysemous nouns have been annotated with the appropriate senses of WordNet 2.0, so the size of the schema (*i.e.*, the total number of senses of these 1,800 words) likely numbers in the thousands; however, they note that this is different from their ontological annotation, for which only approximately 50 concept types are being used to subsume the annotated word senses.

^m^In addition to ~58,000 annotated verbs, OntoNotes has an unstated but presumably large count of annotated nouns.

A summary of counts of words/tokens, of counts and types of component documents, of domains, and of counts of concept annotations for the CRAFT Corpus and related corpora.

Corpora can also be compared on the size of the documents annotated, also summarized in Table 3. Most of the corpora surveyed here are composed of relatively short documents. Among the shortest are those documents that are individual sentences, which compose the GENETAG, the ABGene Corpus, and BioInfer corpora. Most comparable corpora are composed of documents of several sentences to a paragraph, typically publication abstracts, *e.g.*, the CALBC corpus, GENIA, the PennBioIE Oncology and CYP Corpora, GREC, and the Yapex Corpus, as well as those composed of discharge summaries, *e.g.*, the Fourth i2b2/VA Challenge Corpus. The CLEF Corpus is composed of a number of different types of moderately sized medical documents, and the OntoNotes corpus contains 1,000 multiparagraph newswire documents. The longest documents of these surveyed corpora are full-length biomedical articles, *e.g.*, the ITI TXM PPI and TE Corpora, the FetchProt Corpus, and the CRAFT Corpus. In the biomedical domain, having access to full-length articles is increasingly seen as important for concept-identification and information-extraction efforts [57-60].

Another point of comparison of annotated corpora is in terms of their respective domain(s), also summarized in Table 3. The corpora surveyed are within the biomedical domain, with the exception of OntoNotes, which covers English and Chinese newswire text. The CLEF Corpus and the i2b2/VA Challenge Corpus contain clinical documents, which are relatively rare due to issues of patient confidentiality of medical records. The remainder of the corpora discussed here are composed of sentences, abstracts, or full-length articles culled from MEDLINE. However, most of these are further narrowed to one or several relatively specific biomedical domains. In addition to requiring open licensing, the articles of the CRAFT Corpus were selected for their being evidential sources for one or more GO and/or MP annotations of mouse genes or gene products. Apart from focusing on the laboratory mouse (though not exclusively, as evidenced by the unique-concept statistics for the NCBI Taxonomy annotations, as seen in Table 2), the articles have no predefined constraints within the biomedical domain, and the corpus includes articles ranging over the disciplines of genetics, biochemistry and molecular biology, cell biology, developmental biology, and even computational biology. While our corpus does not include examples of articles that do not support GO and/or MP annotations of mouse genes/gene products, *e.g.*, clinical studies, it otherwise reflects a broad overview of the biomedical literature. Compared to other publicly available corpora, CRAFT is a less biased sample of the biomedical literature, and it is reasonable to expect that training and testing NLP systems on CRAFT is more likely to produce generalizable results than those trained on narrower domains. At the same time, since our corpus primarily concentrates on mouse biology, we expect our corpus to exhibit some bias toward mammalian systems.

One of the most important aspects of the semantic markup of corpora is the total number of concept annotations, for which we have provided statistics in Table 3. The full corpus contains over 140,000 annotations to terms from ontologies and other controlled terminologies; the initial release contains nearly 100,000 such annotations. This is among the most extensive concept markup of the corpora discussed here for which we have been able to find such counts, including the ITI TXM PPI and TE corpora, GENIA, and OntoNotes, and it is considerably larger than that of most corresponding previously released corpora, including GENETAG, BioInfer, the ABGene corpus, GREC, the CLEF Corpus, the Yapex corpus, and the FetchProt Corpus. The only corpus with amounts of concept markup considerably larger than ours (and for which we have been able to find such data) is the silver-standard CALBC corpus.

A significant difference between the CRAFT Corpus and many other corpora is in the size and richness of the annotation schemas used, *i.e.*, the concepts that are targeted for tagging in the text, also summarized in Table 3. Some corpora, including the ITI TXM Corpora, the FetchProt Corpus, and the CALBC corpus, used large biomedical databases for portions of their entity annotation, though most were done in a limited fashion.; furthermore, though such databases represent large numbers of biological entities, the records are flat sets of entities rather than concepts that themselves are embedded in a rich semantic structure. There has been a small amount of corpus annotation with large vocabularies with at least hierarchical structure, among these the ITI TXM Corpora and the CALBC corpus, though these are limited in various ways as well. OntoNotes, the GREC, and BioInfer use custom-made schemas whose sizes number in the hundreds, while most annotated corpora rely on very small concept schemas. In the CRAFT Corpus, all concept annotation relies on extensive schemas; apart from drawing from the ~7,200,000 records of the Entrez Gene database, these schemas draw from ontologies in the Open Biomedical Ontologies library, ranging from the ~800 classes of the Cell Type Ontology to the ~410,000 concepts of the NCBI Taxonomy. The initial 67-article release of the CRAFT Corpus contains over 4,300 distinct concepts from these terminologies. Furthermore, the annotation of relationships among these concepts (on which work has begun) will result in the creation of a large number of more complex concepts defined in terms of these explicitly annotated concepts in the vein of anonymous OWL classes formally defined in terms of primitive (or even other anonymous) classes [61]. Analogous to research done in calculating the information content of GO terms by analyzing their use in annotations of genes/gene products in model-organism databases (and from this, the information content of these annotations) [62,63], the information content of biomedical concepts can be calculated by analyzing their use in annotations of textual mentions in biomedical documents (and from this, the information content of these documents).

A crucial difference between the CRAFT Corpus and many other gold-standard annotated biomedical corpora is that markup of concepts requires semantic identity. By this we mean that every annotation in CRAFT is tagged with a term from an ontology or controlled vocabulary such that the text selected for the annotation is essentially semantically equivalent to the term; that is, each piece of annotated text, in its context, has the same meaning as the formal concept used to annotate it. In many other corpora, text is marked up even if the concept denoted is more specific than the concept used to annotate it; this approach is sometimes referred to as marking up all mentions “within the domain of” the given annotation class. For example, given a schema with a cell class (but nothing more specific), most corpora would annotate a mention of the word “erythrocyte” to that class. This results in semantic loss: It is not the case that the annotated text means the same thing as the associated semantic class. The size of the annotation schemas and the principle of semantic identity make assertions involving annotated concepts more valuable. For example, if the goal is to identify specific proteins expressed in specific cell types, annotations to generic categories such as “protein” or “cell” are not adequate.

Though it may sound straightforward to mark up all mentions of a given annotation class, it is often difficult and can seem subjective. Tateisi *et al.* have reported on the difficulty of distinguishing the names of substances from general descriptions of the substances in the construction of GENIA [64], and there was relatively low agreement on what qualified as, *e.g.*, activators, repressors, and transcription factors in the GREC [50]. This is even more difficult when it involves identifying precise text spans for annotation. Our annotators found that evaluating whether a span of text is semantically equivalent to a given term is easier than attempting to evaluate whether a piece of text refers to a concept that is subsumed by a more general schema class but not explicitly represented. It is for this reason that we emphasize annotation to an ontology/terminology rather than to a domain. Domain boundaries are often ill-defined, which makes it difficult to evaluate whether a piece of text refers to a concept that “should be” in some ontology; thus, we annotate only to what actually is in an ontology, not to some abstract idea of its domain. For example, if the ontology being used to annotate the corpus contains a concept representing vesicles but nothing more specific than this, a textual mention of “microvesicle” would not be annotated, even though it is a type of vesicle; this is because this mention refers to a concept more specific than the vesicle concept (and our annotation guidelines do not allow annotations to a part of a word such as this). In other cases, a portion of a mention to a concept missing from an ontology can be marked up; for example, for the text “mutant vesicles”, “vesicles” by itself is tagged with the vesicle concept. We regard such an approach as a strength, as only text that directly corresponds to concepts represented in the terminology is selected. Although experts might use such texts to make suggestions of new concepts to ontology curators, such activity was in general beyond the scope of the annotation work itself. However, we expect that the CRAFT Corpus could be exploited by ontology curators to find such missing concepts.

The CRAFT Corpus is distinguished by the quality and applicability of the schemas (*i.e.*, potential target concepts) used for annotation. Many other corpora rely on concept schemas custom-made for their specific projects, often with representational idiosyncrasies; such schemas are not widely reusable for other purposes. Some corpora, such as the GREC and the event subset of GENIA, use schemas based, at least in part, on subsets of established external resources. The CRAFT Corpus is unique in that it relies on well-established, independently curated resources in their entirety. Eight of these resources are formal biomedical ontologies developed within the sphere of the Open Biomedical Ontologies (OBO) movement and are dedicated to faithfully representing the concepts within their respective domains, including five in the OBO Foundry that conform to an additional set of ontological principles. By predominantly annotating to widely used, high-quality terminologies, the CRAFT Corpus builds on years of careful knowledge representation work and is semantically consistent with a wide variety of other efforts that exploit these community resources.

In addition to using community-curated resources in our scheme, CRAFT also annotates every mention of nearly^c^ every concept that appears in the texts. Although such an approach seems intuitive (and is clearly beneficial for training machine-learning NLP systems), it is not used in a number of corpora. Tanabe *et al.* have written that “one fundamental problem in corpus annotation is the definition of what constitutes an entity to be tagged” and cited the complex guidelines of the MUC-7 Named Entity Task as evidence [47]. In BioInfer, the focus is the annotation of relationships among genes, proteins, and RNAs, and entities are only annotated if they are relevant to this focus and if they are named entities—a term itself with much baggage, however, if the arguments of primary events are other events or qualities that recursively have genes, proteins and/or RNAs as arguments, these secondary events or qualities are annotated as “extended named entities”, but they are annotated only in such cases. In the PennBioIE Oncology corpus, a gene is only annotated if there is an associated variation event, and in the i2b2/VA Challenge corpus, only concepts lexicalized as complete noun phrases are annotated; *e.g.*, “diabetes” is annotated in “she developed diabetes” but not in “she takes diabetes medication”.

The span selection guidelines for the concept annotations of the CRAFT Corpus also provide important advantages. Given an initial anchor word as the basis for an annotation, the rules for deciding which adjacent words can be considered for inclusion in an annotation and which cannot are precise and purely syntax-based, and the decision as to whether to include one or more modifiers or modifying phrases rests solely on whether their inclusion would result in a direct semantic match to a concept in the terminology being used. Unlike some other corpora (*e.g.*, GENETAG, the ITI TXM corpora), annotations in CRAFT can be discontinuous, *i.e.*, can be composed of two or more nonadjacent spans of text, though these must still abide by the same span-selection guidelines. Use of discontinuous annotations allows us to ensure that only text that is semantically identical to a concept is marked, regardless of internal interruptions. In some corpora, there are unclear guidelines (and consequently inconsistent annotations) for the text spans associated with an annotation. For example, in GENIA, “the inclusion of qualifiers is left to the experts *sic* judgment” for the task of entity annotation [48], and in the i2b2/VA Challenge corpus, “[u]p to one prepositional phrase following a markable concept can be included if the phrase does not contain a markable concept and either indicates an organ/body part or can be rearranged to eliminate the phrase” [46]. The CRAFT specifications minimize subjective selections, and increase interannotator agreement on spans. CRAFT text span-selection guidelines are quite extensive (see supplementary materials), but our biomedical-domain-expert concept annotators with no previous experience with formal linguistics were able to quickly learn them.

Finally, few corpora have attempted to capture semantic ambiguity in concept annotations. The most prominent way in which CRAFT represents concept ambiguity is in cases in which a given span of text could be referring to two (or more) represented concepts, none of which subsumes another, and we have not been able to definitively decide among these. This occurs most frequently among the Entrez Gene annotations, in which many mentions of genes/gene products not grammatically modified with their organismal sources are multiply annotated with the Entrez Gene IDs of the species-specific genes/gene products to which these mentions could plausibly refer. Similar to GENIA, this multiple-concept annotation explicitly indicates that these cases could not be reliably disambiguated by human annotators and therefore are likely to be particularly difficult for computational systems. Explicitly representing this ambiguity allows for more sophisticated scoring mechanisms in the evaluation of automatic concept annotation; for example, a maximum score could be given if a system assigned both insertion concepts to the aforementioned example and a partial score for an assignment of only one of these concepts. . However, we have attempted to avoid such multiple annotation by instead singly annotating such mentions according to improvised guidelines for specific markup issues (which do not conflict with the official span-selection guidelines but rather build from them). For example, some nominalizations (*e.g.*, insertion) may refer either to a process (*e.g.*, the process of insertion of a macromolecular sequence into another) or to the resulting entity (*e.g.*, the resulting inserted sequence), both of which are represented in the SO, and it is often not possible to distinguish among these with certainty; we have annotated such mentions as the resulting sequences except those that can only (or most likely) be referring to the corresponding processes. A simpler case involves a text span that might refer to a concept or to another concept that it subsumes. In such a case, only the more general concept is used; for example, *Mus* refers both to a organismal-taxonomic genus and to one of its subgenera, so a given mention would only be annotated with the genus; the rationale for this decision is that it is generally not safe to assume that the more specific concept is the one being mentioned.

### Ongoing and future work

In addition to the conceptual annotation that is described here and the syntactic annotation that we describe in a companion article [27], there are multiple ongoing projects that add additional layers of annotation to the CRAFT Corpus data, all of which will be made available in future releases of the corpus:

· We have begun work on assertional annotation of the corpus, i.e., the markup of assertions among the annotated concepts by linking them via relations. We have encountered many difficult aspects in this task, which may be challenging to accomplish as consistently as the concept annotation. We seek to create this assertional markup using a methodology such that the annotations will be able to be programmatically translated into formal knowledge representations that can be stored and queried in an RDF knowledge base [61].

· An extensive project is nearly complete to mark all coreference in the corpus. The two relations of COREF (coreferentiality) and APPOS (appositive) are marked. The guidelines for this portion of the work were adapted from the OntoNotes guidelines, with the major difference that we did not utilize the category of generics. As we have discussed in relation to the guideline selection process for this task [28], we maintain that in the biomedical domain, in which everything mentioned, including abstract concepts such as data, belongs in the domain of an ontology, the notion of genericity does not apply.

· Discourse annotation on the sentence level, using the CISP/ART schema [65], is nearly complete. An early result of this work has been the finding that sequences of rhetorical moves can be characterized by finite state machines.

· The contents of all parentheses are being annotated with respect to a schema of twenty categories, including citations, data values, p-values, figure/table pointers, list elements, and others. We have previously presented the annotation procedure and the use cases for the various categories in the schema, as well as a classifier for determining category membership of contents of parentheses [66].

· As a primary criterion in the selection of articles for the corpus was their use as evidential sources for ontological annotations of mouse genes/gene products in the Mouse Genome Database (a major component of the Mouse Genome Informatics resources), we have marked up the specific sentences within these articles upon which these annotations are based. Motivated by a growing need for semiautomatic assistance in the curation of data in model-organism databases, we intend for this to serve as a gold standard for the training of systems to identify relevant evidential sentences in the biomedical literature.

Furthermore, in the future, we intend to periodically update the annotations using current versions of the OBOs as well as correct errors that we find or are brought to our attention.

## Conclusions

The concept annotation of the CRAFT Corpus, a collection of 97 full-length, open-access biomedical journal articles, is designed to serve as a high-quality gold standard for the training and testing of advanced biomedical NLP systems. In our corpus, we have created annotations for all mentions of nearly all concepts from nine prominent biomedical ontologies and terminologies, consistently created based on one set of guidelines. CRAFT displays consistently high interannotator agreement, as evaluated by single-blind review by the lead semantic annotator of the primary annotators’ markup.

At approximately 560,000 tokens in the initial 67-article release and 830,000 tokens in the full set, the CRAFT Corpus is among the largest gold-standard annotated biomedical corpora, and unlike most others, the journal articles that comprise the documents of the corpus cover a wide range of biomedical disciplines. Additionally, with a total concept annotation count of nearly 100,000 in the initially released 67-article subset and of over 140,000 in the full collection, the scale of our conceptual markup is also among the largest of all comparable corpora. Along with the syntactic and coreferential annotations that have been created for the same set of journal articles, the concept annotations of the CRAFT Corpus have the potential to significantly advance biomedical text mining by providing a high-quality gold standard for NLP systems.

## Methods

### Corpus assembly

The 97 articles of the corpus were selected based on (a) their use by the Mouse Genome Informatics (MGI) group [67], each of which was used as an evidential source for one or more annotations of mouse genes or gene products in the Mouse Genome Database (MGD) [68] to one or more terms from the GO and/or the Mammalian Phenotype Ontology (MP) [69], and (b) for their unrestrictive licensing terms, *i.e.*, available in PubMed Central in the form of Open Access XML. Table 4 shows counts for each category; for example, 7,263 articles were used as the evidential sources for MGI annotations using only GO terms; of these, 1,249 were available in PubMed Central, and of these, only 27 were available in PubMed Central in the form of Open Access XML. Note that although the last column adds up to 98, one of these articles was not available in its full-text form at the time the corpus was being assembled and was thus excluded from it. The 67 articles of the initial release set were selected on the basis of their being representative of the entire corpus in terms of distribution of concept annotations. One-way ANOVA statistics were calculated for each terminology used to annotate the corpus, and based on these tests, the release and test sets were shown to not be statistically different in terms of these concept-annotation distributions [27].

**Table 4:** Statistics for MGI annotations and articles

**Ontology/Ontologies Used in Annotation**	**# Articles Cited as Evidential Sources**	**# Articles Cited as Evidential Sources & Available in PubMed Central**	**# Articles Cited as Evidential Sources & Available in PubMed Central as Open Access XML**
GO (only)	7,263	1,249	27
MP (only)	10,469	2,699	66
GO & MP	2,174	633	5

Statistics for MGI annotations and articles for the assembly of the CRAFT Corpus.

### Ontology/terminology selection

The annotation of the biological concepts in the corpus was performed using ontologies and other controlled terminologies in their entirety. These ontologies and terminologies were chosen based on their quality and their representation of domain-specific concepts frequently mentioned in biomedical text. As precedence was given for a representation in the form of a well-constructed, community-driven ontology, seven of these (ChEBI, PRO, GO BP, GO CC, GO MF, CL, and SO) are Open Biomedical Ontologies, and the first five of these are OBO Foundry ontologies, indicating an official endorsement of quality by this consortium [7]. In addition, to mark up some essential biological concepts not yet represented in a proper ontology, we chose to use the unique identifiers of the NCBI Taxonomy, as this is the most widely used Linnaean hierarchy of biological taxa, and the unique identifiers of the Entrez Gene database, as this is the most prominent resource for information pertaining to species-specific genes. Details of versions of all of the ontologies and terminologies used as well as their application toward the creation of the concept annotations are presented in the Methodology.

For each annotation pass with an OBO, a version of the ontology at the start date of the annotation pass was frozen so that all of the annotations of a given pass were semantically consistent and relied upon a single ontology version. Though these ontologies have evolved since the start of the project, all of the annotations are stored in terms of their formal IDs, permitting their mapping to concepts in current versions. We have included the versions of the ontologies that were used for annotation in the release package, and we recommend that users rely on these versions when working with the CRAFT Corpus.

### Creation of annotation guidelines

Concept-annotation guidelines were created by the semantic annotation lead (MB), a researcher with extensive experience with biomedical ontologies and knowledge engineering, along with an experienced computational linguist (ME). Initial guidelines were developed through iterative attempts by MB to manually annotate biomedical journal articles and assess whether the proper use of the guidelines consistently resulted in intuitive concept annotations using the terms of the ontology or terminology with subjective decisions kept to a minimum, revising the guidelines accordingly. As presented in the Discussion section, a number of other biomedical concept-annotation projects suffer from a lack of clarity in terms of selection of text spans for the annotations, resulting in increased difficulty of the task and thus decreased performance. To preclude this, it was decided early in the project that span-selection guidelines based strictly on syntax would minimize subjectivity and maximize performance of this task. This required us to develop an extensive set of syntax-directed guidelines covering nouns and noun phrases, adjectives and adjectival phrases, prepositional phrases, relative clauses, appositions, and adverbs, as well as rules for overlapping and nesting of concept annotations, coordinated phrases, and discontinuous annotations. Further minimizing subjectivity in the task, we specified that any such text that is considered for inclusion in a given annotation is included only if its inclusion results in a direct semantic match to a concept in the ontology or terminology being used to annotate the text.

Despite the initial worry that the primary annotators for this task, all of whom had degrees in the biological sciences and no formal experience with linguistics, would have difficulty adhering to the annotation guidelines with their detailed basis in syntax, they quickly achieved proficiency in correct span selection. As they proceeded to annotate the articles, only minor revisions had to be made to the syntactic aspects of the guidelines, and span-selection errors due to nonadherence to these syntactic aspects accounted for a small minority of the errors detected in the regular review of their markup by the semantic lead annotator.

Rather than these syntactic aspects of the guidelines, it was progressively shown that annotation discrepancy between the primary annotators and the reviewing semantic lead annotator was mostly the result of semantic issues in using the ontologies and terminologies for text annotation. These semantic issues can be broken down into two main subtypes: (1) A part of the ontology or terminology being used was insufficiently rigorous in its representation or ill-suited for the task of text annotation, resulting in its inconsistent use; and (2) The primary annotator was not aware of some representational aspect of the ontology or terminology being used. It was these types of semantic issues that required more substantial amounts of time to resolve and for which having a knowledge engineer experienced with these ontologies and terminologies proved extremely valuable.

Upon encountering an instance of the first semantic issue subtype through the review of the primary annotators’ markup, the semantic lead annotator, after reviewing as many annotation instances of the specific issue as could be found, developed informal (and sometimes suboptimal) guidelines for addressing it; this included narrowing the cases in which a set of concepts was to be used and, in rare circumstances, not using a set of concepts at all. These more informal annotation guidelines, specific to these issues, were communicated in the periodic meetings with the primary annotators as they arose, subsequent to which the annotators attempted to follow them. Multiple rounds of this process, with discussions among the primary and lead semantic annotators, were often required until a satisfactory solution with relatively few discrepancies among the annotators was reached. Upon encountering an instance of the second semantic issue subtype, the semantic lead annotator reviewed the relevant portion or representational aspect of the ontology or terminology and its correct use with the primary annotator in one of their periodic meetings, along with similar followup to ensure that the latter was subsequently using these concepts correctly.

Due to space restrictions, we include a discussion of our concept-annotation guidelines, which were used for all nine terminological annotation passes, as supplementary material to this paper; alternately, the reader may refer to our previously published paper presenting the guidelines [70] or to the guidelines in their entirety on the project Web site (Additional file 2: Doc2). They are freely available for external use under a Creative Commons license.

### Creation of markup

The annotation of the articles with each ontology and terminology was performed independently with the exception of the GO BP and MF ontologies, which were used to mark up the text concurrently in one pass. With few exceptions, every mention in the articles of the corpus of every concept of the ontologies and terminologies has been annotated with the appropriate concept(s), and the guidelines presented as supplementary material were used for all of the concept-annotation passes. In an attempt to reduce the workload of the human annotators, most of the annotation passes using an OBO relied on an initial preprocessing to programmatically create an initial set of annotations of concept mentions appearing as exact matches or plurals of names or exact synonyms of the terms of the OBO.

There was no prior training on other texts; after a short introduction to the guidelines and to the annotation tool, the annotators began their work directly on the articles of the corpus. For each of the nine conceptual passes, the corpus with its corresponding programmatically created annotations was then reviewed by a primary human annotator. All of this primary manual markup was performed by three annotators, each of whom has a Ph.D. in the biological sciences, and the annotator for the GO BP & MF passes also served as the primary annotator of GO annotations of mouse genes and gene products for the MGD. Entailed within this review was the checking of each of the preliminary annotations in terms of its span (*i.e.*, the set of characters selected) and its class (*i.e.*, the term with which this set of characters is annotated), making revisions to either or both of these or deleting the entire annotation as assessed. Furthermore, the annotator examined the entire text of each article, creating annotations missed by the programmatic pass. These annotators searched for concepts among the ChEBI, CL, GO BP/CC/MF, PRO, and SO ontologies directly in the Protégé-Frames interface, as each of these ontologies was imported into the corresponding annotation project, while search for concepts in the Entrez Gene and NCBI Taxonomy databases was performed via their Web interfaces. In each case, the annotator searched for concepts using their entire names, synonyms, and then subsets of these, as progressively required. The Protégé-Frames, Entrez Gene, and NCBI Taxonomy interfaces all allow for searching for subsets of concept names, and all return partial matches of inputted query phrases; searching for existing concepts among the ontologies and terminologies was not a significant difficulty.

On an approximately weekly basis, all of these resulting annotations were sent to the semantic lead annotator, who subsequently checked each annotation from the most recent set of annotations in terms of its span and class, noting any disagreement with the primary manual markup, and also read through the text to find any possibly missing annotations. For each of these annotation sets, a meeting by telephone was conducted between the primary and semantic-lead annotators, and any annotation disagreements between the primary and the semantic lead annotators were discussed until a consensus was reached, which sometimes resulted in further changes in guidelines; the markup was appropriately modified to reflect this consensus. IAA statistics were calculated for each annotation time period.

Knowtator [37], which is implemented as a tab plugin to Protégé-Frames [71], was used for all semantic-annotation work. Initial training sessions were conducted face to face (with the exception of the GO BP & MF annotator, who was not situated in the local area) until the annotator gained a reasonable facility with the tool and task. Each annotator sent his or her Protégé project (which contains all of the annotations) to the semantic lead on an approximately weekly basis, and, following the initial training period, an annotation meeting was conducted individually by phone after the lead reviewed his or her most recent markup through the Protégé project. All IAA statistics were calculated through Knowtator.

#### Cell type ontology (CL)

For the annotation of cells, we used the 1.26 version of the CL dating to 2007 05 25, which contains 838 terms. The CL has a cell line cell term (CL:0000010) but no specific types of cell-line cells, so these specific types are not annotated in the corpus. (The Cell Line Ontology [72] would be useful for this task as future work on the corpus.)

#### Chemical entities of biological interest ontology (ChEBI)

The annotation of the corpus with ChEBI relied upon release version 45, dating to 2008 05 28, which contains 19,633 terms representing types of biochemical roles and applications, subatomic particles, atoms, molecules and other polyatomic entities, and their parts (*i.e.*, groups). Mentions of elements without specification of charge have been annotated with terms from the branch of atoms, while those with specification of charge have been annotated with terms from the branch of elemental molecular entities; though these branches are not integrated with each other (as we believe they should), this protocol allows for the closest semantic matches. Mentions of polyatomic ions without specification of charge are multiply annotated if there is no corresponding charge-independent ChEBI concept; *e.g.*, “glutamate” is doubly annotated with glutamate(1-) (CHEBI:14321) and glutamate(2-) (CHEBI:29987), as there is no more general term for glutamate without specification of charge. There are a number of ChEBI concepts representing types of biological sequences in their full molecular forms that were challenging to use because many textual sequence mentions are ambiguous as to whether they refer to full molecules or to proper subsequences, specifically deoxyribonucleic acids (CHEBI:16991), ribonucleic acids (CHEBI:33697), oligonucleotides (CHEBI:7754), dinucleotides (CHEBI:47885), peptides (CHEBI:16670), oligopeptides (CHEBI:25676), dipeptides (CHEBI:46761), tripeptides (CHEBI:47923), tetrapeptides (CHEBI:48030), and pentapeptides (CHEBI:48545). Since this ambiguity is captured in our annotation of these mentions with cognate concepts in the sequence ontology, these more specific ChEBI concepts were not annotated. Annotating nested parts of mentioned polyatomic entities has been challenging, as they often can plausibly refer to multiple concepts; *e.g.*, “amino” of “amino acid” could refer to amine or amino group, which are both represented in the ontology (and in different branches); though we have annotated all such nested ChEBI concepts, we recommend not attempting to mark up ChEBI concepts nested within other ChEBI concepts when annotating biomedical text, as this would render many of these moot. Finally, text was not marked up with label (CHEBI:35209) or tracer (CHEBI:35204), as these concepts were found difficult to use in practice.

#### Entrez gene (EG)

The identification of genes and gene products in text has been a primary focus of biomedical text mining, and the difficulties encountered in marking up mentions of these entities (*e.g.*, [45,73]) broadly fall into two categories: ambiguity of species/taxon and ambiguity of sequence type. As for the former, one of the most difficult aspects of markup up mentions of genes and their derived sequences has been determining whether a given mention referred to a species-specific entity, an entity corresponding to a higher-level biological taxon (*e.g.*, mammalian CLN2), or to a taxon-independent entity. Since all of the entries of the Entrez Gene database are species-specific, only the mentions of the first type can be annotated with Entrez Gene entries at all. Unfortunately, it is often not possible to reliably choose among these options; authors themselves seem to conflate these types and/or jump from one framing to another, and more than one of these options often fits for a given mention. The CRAFT Corpus employs a fairly liberal approach by marking up a given sequence mention with a given Entrez Gene ID if it is plausible—not certain—that the authors are referring to the species-specific sequence denoted by the ID; in addition, the identity of the species of the given sequence must be mentioned in the article itself. With these criteria, the large majority of mentions of genes and their derived sequences could be annotated with Entrez Gene IDs. Many of these are annotated with multiple IDs; this indicates, for a given mention, that the authors may be referring to any of multiple organisms mentioned in the article. Mentions of genes and their derived sequences that are not marked up with Entrez Gene IDs include (a) those that are found in general/background statements; (b) those whose organismal source is not mentioned in the respective journal article, including those with citations in which the source can only be determined by examining the cited publication(s); and (c) those that do not have corresponding Entrez Gene entries, particularly genes and gene products used in experiments that are not the focus of the articles' research (*e.g.*, restriction enzymes).

The other primary vexing aspect of this task is the determination of sequence type, an issue that also has been encountered in other markup efforts. The difficulty in specifying whether a given mentioned sequence refers to a gene, a transcript, or a polypeptide is well-known, but we have also found mentions of sequences denoted by Entrez Gene records that actually refer to homomeric complexes, promoters, enhancers, pseudogenes, cDNAs and quantitative trait loci, among others. In addition to the aforementioned specification of Entrez Gene IDs, we initially marked up these mentions with regard to sequence type as well, using ontological terms, principally from the SO, *e.g.*, gene (SO:0000704). However, this task grew increasingly problematic, and we decided to mark up these mentions only with regard to Entrez Gene ID. Therefore, all such mentions are annotated to a generic Entrez Gene sequence class, and the Entrez Gene ID is specified in the has Entrez Gene ID field. Furthermore, these annotations have been created without regard to sequence type: Not only are genes annotated, but transcripts, polypeptides, and other types of derived sequences are equivalently marked up with the Entrez Gene IDs of their corresponding genes. Thus, an Entrez Gene annotation refers to the DNA sequence denoted by the Entrez Gene record *or* to some sequence derived from it.

Even though we have removed the ambiguity with regard to sequence type, the Entrez Gene annotations could still prove challenging to use due to the aforementioned ambiguities of whether to mark up a given mention or to regard it as a more general mention and, if it is to be marked up, which one or more species-specific sequence versions to use to mark it up. These were difficult issues even for us as manual annotators, and we expect that they would be even more difficult for computational systems. We believe that there are no easy solutions to marking up these sequence mentions with a species-specific vocabulary such as the Entrez Gene database and that a vocabulary that includes taxon-independent sequences should instead be used for conceptual annotation of these mentions. We have also marked up mentions of sequences with the PRO (detailed below), which includes taxon-independent sequence concepts (on which we relied), and we recommend that researchers use the PRO annotations rather than the Entrez Gene annotations for identification of genes and gene products in biomedical text, as we are more confident of the consistency and utility of the former than the latter.

#### Gene ontology biological processes (GO BP)

For the annotation of biological processes, we used the 1.9 revision of the GO dating to 2007 11 28, which contains 14,306 BP terms; these processes span a wide range of granularities, including those at the level of molecules, subcellular structures, cells, tissues/organs, and organisms and even extend to multiorganismal processes. This pass accounts for the large majority of verb-based annotations, and it is the most syntactically complex set of annotations, including many with prepositional phrases and adjectives/adjectival phrases as well as many discontinuous annotations. For many of the annotations, a word is marked up with a biological process given the context even though the word may be used in a more general sense in another context, *e.g.*, expression, differentiation, and proliferation. Many vexing semantic issues, small and large, arose during the course of the project [31,32]. For example, there are many mentions of development/production/generation/synthesis/formation processes; depending on what is being generated, these may be, *e.g.*, biosynthetic processes (for molecules and parts of molecules), cellular component assemblies (for cellular components), reproduction (for organisms), or developmental processes (for anatomical parts)--which are all separate branches of the ontology. The last of these is further complicated by the fact that there are three prominent subhierarchies of developmental processes (those for formation, morphogenesis, and development) that have very specific definitions that by and large do not conform to the mostly interchangeable textual use of “develop” and its synonyms. For mentions of developmental processes, we have used the appropriate development term if there is such a term for the anatomical structure mentioned in the selected text, the appropriate formation term if there is a corresponding formation term (and no development term) for the structure, a morphogenesis term for any explicit mention of morphogenesis (as this concept is unambiguous), and the top-level BP term developmental process (GO:0032502) if there is no corresponding term in the ontology for the structure and/or if the structure is not mentioned within the selected text. A relatively broad set of words have been considered semantically very close for biological regulation (*e.g.*, "monitor", "govern", "control", "guide"), positive regulation (*e.g.*, "promote", "augment", "enhance"), and negative regulation (*e.g.*, "impair", "repress", "suppress"), so these words (along with their lexical variants) are annotated relying on these concepts in appropriate contexts. However, some were considered semantically narrower than these (*e.g.*, "activate", "trigger", and "induce" for positive regulation and "block", "inhibit", and "inactivate" for negative regulation) and thus were not annotated relying on these concepts.

#### Gene ontology cellular components (GO CC)

The annotation of the corpus with terms of the GO CC ontology relies on the aforementioned version of the GO, which contains 2,047 CC terms representing subcellular structures, both intracellular and extracellular. This pass of the annotation is mostly straightforward. One difficulty concerns chromosomal part (GO:0044427) in that though a DNA subsequence (*e.g.*, a gene, a QTL) is a part of a chromosome, these subsequences are not included in this ontology under chromosomal parts, but authors do refer to these subsequences as chromosomal regions; we have attempted to disambiguate the nature of mentions of chromosomal regions, but it is somewhat subjective without a specification of the relation between the GO CC concept chromosomal part (GO:0044427) and the SO concept region (SO:0000001). Additionally, this ontology also contains classes representing macromolecular complexes (which we have previously argued should not be part of this ontology) that have proven difficult: Often it is unclear whether a given mention of a macromolecular entity is a macromolecular complex (in which case it does refer to the GO CC concept) or a single macromolecule (in which case it does not); an example of this are mentions of receptors, which may be either single proteins or protein complexes, the former of which do not refer to receptor complex (GO:0043235). It is often difficult to ascertain whether the type of mentioned receptor can form a complex and if so, if it is doing so in a particular context; this is even more ambiguous if multiple types of receptors are being discussed or if the types of receptors are not specified. Assuming there is a GO CC macromolecular-complex term to which a given mention may refer, a mention is straightforwardly annotated if it is clearly specified as a complex, *e.g.*, “receptor complexes”. If there is no such clear specification, it is annotated if the mention is also the name of a protein that may be in the form of a homomeric complex in its context (*e.g.*, tubulin complex (GO:0045298) for “tubulin”) *except* if there is a corresponding MF term (*e.g.*, receptor activity (GO:0004872) for “receptor”). If there is such a corresponding MF term, the mention is *not* annotated with the CC term, since this ambiguity can be captured using the MF term and the often-tricky issue as to whether to regard and annotate such as a mention as a macromolecular complex can be avoided.

#### Gene ontology molecular functions (GO MF)

As the annotation of GO molecular functions was performed simultaneously with the GO biological processes by the same annotator, the aforementioned version of the GO was used, which contains 7,984 MF terms; among the functions represented by these terms are types of binding, transporter activity, molecular transducer activity, and catalytic activity. We have previously written of the difficulty of distinguishing among and annotating with GO BP and MF concepts in text [31,32], and these issues have continued to make consistent annotation of text with GO MF concepts in particular challenging. As a suboptimal solution, we have narrowly annotated the articles of the corpus with the GO MF terms. The majority of these annotations identify molecular entities possessing the specified functionalities, and the text spans of these annotations are additionally marked up with independent_continuant (snap:IndependentContinuant^d^); so, for example, the annotation of “cation channel” with the GO MF concept cation channel activity (GO:0005261) and also with snap:IndependentContinuant has the semantics that this text span refers to an independent continuant that has cation channel functionality. The one primary subgraph of the GO MF ontology whose terms are predominantly annotated as molecule-level processes rather than as molecular entities possessing functionalities is the binding (GO:0005488) hierarchy.

#### NCBI taxonomy (NCBITaxon)

As with the annotations with the unique IDs of the records of the Entrez Gene database, annotators working with the NCBI Taxonomy directly used the NCBI Taxonomy interface [75] to search for entries denoting organisms. The difficulties in ontological representation of biological taxa has been discussed elsewhere [76,77]; for this project, we have regarded the entries of the NCBI Taxonomy database as denotations of organisms since its taxonomic relationships hold for organisms (*e.g.*, a rodent is a kind of mammal) but possibly not for the taxa themselves. (For example, it is not clear that the order *Rodentia* is a kind of the class *Mammalia*.) As with all other projects, the closest semantic match was used; thus, a mention of "rat" (and not more specific than this) is marked up with Rattus (NCBITaxon:10114), which has common names of "rat" and "rats" in the database, even if from context it is known to be, *e.g.*, the common laboratory rat *Rattus norvegicus*. The terms of the other sequences (NCBITaxon:28384) and unclassified sequences (NCBITaxon:12908) subtrees were not used for markup, as we felt they were of dubious quality and relevance. Mentions of lexical variants of top-level words such as "organism" and "individual" are annotated with the root node of the named taxa, root (NCBITaxon:1). In order to differentiate mentions of organisms (*e.g.*, "rat") from mentions of taxa denoting these organisms (*e.g.*, "*Rattus*"), the latter are additionally annotated with the term taxonomic_rank (NCBITaxon:taxonomic_rank). For mentions of taxa that have identical lexicalizations (*e.g.Xenopus* denotes both a genus and a subgenus), the more general one is used. Finally, mentions of taxonomic ranks themselves (*e.g.*, class, family, species) are annotated with the appropriate terms of the taxonomic_rank subtree.

#### Protein ontology (PRO)

The annotation of the corpus with the PRO relied on the 2011 04 22 version of the ontology. Even though this ontology focuses on proteins (and to a small extent protein complexes), the articles of the corpus are marked up with PRO annotations without regard to sequence type, as with the Entrez Gene annotations. For example, all “NT-3” sequence mentions are annotated with neurotrophin-3 (PR:000011459) whether a given mention refers to a gene, a transcript, a polypeptide, or some other type of derived sequence; thus, the implied semantics of such an annotation encompasses this range of sequence types. Even in a case in which the sequence type is explicitly stated, the sequence type is not included in the annotation (also as in the Entrez Gene annotations); for example, for a mention of “NT-3 mRNA”, “NT-3” alone is marked up with neurotrophin-3. This use of the PRO has worked well in conjunction with the use of the SO (see below), as most of these explicitly stated sequence types are captured in SO annotations.

Most of the protein concepts of the PRO are taxon-independent, an attribute that has greatly simplified the annotation of these specific sequence mentions as compared to the task of their annotation with the entries of the Entrez Gene database (see above). In some cases, these taxon-independent protein concepts are subclassed with species-specific version; for example, the taxon-independent delphilin (PR:000008239) is subclassed with delphilin (mouse) (PR:000025479), defined in terms of *Mus musculus*. However, these were seldom used, as even a given sequence mention that explicitly states a taxon is typically not explicitly species-specific. For example, a mention of “mouse delphilin” would *not* be annotated with delphilin (mouse) because the mention only explicitly states “mouse”, whose closest semantic match is the genus *Mus* (in concordance with our NCBI Taxonomy annotations, see above), whereas delphilin (mouse) is formally defined in the ontology in terms of *Mus musculus* (even though it only specifies “mouse” in the name). Thus, delphilin (mouse) is too taxonomically specific for this mention, and only “delphilin” of “mouse delphilin” would be annotated with the taxon-independent delphilin. However, a mention of “*Mus musculus* delphilin” *would* be annotated with delphilin (mouse), as this would now be a direct semantic match.

Because of the presence of the taxon-independent protein concepts in the PRO, we were able to annotate many of the sequence mentions that we were not able to annotate with Entrez Gene entities, including those referring to sequences without regard to taxa, those whose species identities are only indicated in cited articles or other resources, and those referring to higher-level taxa. Furthermore, most of the sequence mentions that are annotated with multiple Entrez Gene entities due to species ambiguity are more straightforwardly annotated with single taxon-independent PRO concepts. We are more confident of the consistency and utility of the PRO annotations than the Entrez Gene annotations, and we recommend using the former for identification of specific genes and gene products in text.

It should be noted that the PRO ontology file contains concepts from other ontologies (including the GO, ChEBI, and NCBI Taxonomy), which are used for classification and formal definition of PRO concepts. However, we did not use any of these concepts from other ontologies in the PRO annotation pass, as they are not PRO concepts, even though they appear in the ontology file. Therefore, we recommend that users ignore these concepts (which have namespace prefixes other than the PRO prefix “PR”) when using the PRO ontology file (which is included in the release package, along with all of the other versions of the ontologies that were used) to annotate text.

#### Sequence ontology (SO)

The annotation with the SO used the 1.45 revision of the ontology, dating to 2009 03 30, which contains 1,610 terms representing types of biomacromolecular sequences, their attributes, and processes of sequence variation. This set of annotations is very large considering the relatively small size of the ontology; this can be accounted for by the very large number of mentions of basic sequence types such as genes, proteins, alleles, chromosomes, and genomes in these articles, all of which are annotated with SO concepts.

This is the only ontology used in this project that contains represented attributes, *e.g.*, flanked (SO:0000357) and linear (SO:0000987). While some of these have been straightforward to use and mainly applied to adjectives, others have not, which necessitated strategies other than attempting the often-difficult task of classifying a given mention as a reference to a sequence attribute or to a sequence itself. Other than flanked, sequence-attribute concepts lexicalized as past participles, particularly those classified under gene_attribute (SO:0000401) (*e.g.*, regulated (SO:0000119)) and transcript_attribute (SO:0000237) (*e.g.*, polyadenylated (SO:0000246)) were not used, as such mentions were already being annotated as references to corresponding GO biological processes (see above). The attributes enzymatic (SO:0001185), peptidyl (SO:0001407), nucleic_acid (SO:0000348), and all of its subclasses were treated as independent entities rather than properties, and so all mentions of these in text, modifying or not, are annotated; for example, all mentions of “peptide” are annotated with peptidyl whether they modify other sequence words or not. The concept transgenic (SO:0000781) was not used at all, instead annotating all transgene mentions, modifying or not, with the corresponding independent entity transgene (SO:0000902).

If not modifying sequences or biological entities containing sequences, textual mentions annotated with wild_type (SO:0000817) are also annotated with independent_continuant (see annotation with GO MF, above) to indicate that this refers to some unmentioned type of entity with some specified wild-type sequence. For example, for “as seen in the wild-type”, where “wild-type” refers to organisms with some specified wild-type sequence, “wild-type” would also be annotated with independent_continuant. Similarly, where “transgenic” indicates unmentioned organisms, it is annotated with both transgene and independent_continuant; the same applies to sequence_alteration where, *e.g.*, unmentioned mutant organisms are being referenced. However, users may want to ignore these independent_continuant annotations and not attempt to distinguish these cases, as this is a tricky ontological issue.

This version of the SO contains basic types of sequence variations and their corresponding processes (*e.g.*, substitution (SO:1000002) and substitute (SO:0000048)) that are often challenging to differentiate as nominalizations in text. We have decided to annotate such a nominalization as the former except where it is reasonably clear that it is referring to the process, particularly when the nominalization is not preceded by an article, *e.g.*, “by substitution”. Finally, the concepts of the sequence_variant_effect (SO:1000132) and chromosome_variation (SO:0000240) subgraphs of the SO were not used, as their representation and definition were problematic, which hindered their use toward annotation. However, we have begun collaborative work with the developers of the SO toward an improved representation of sequence variation [78], along with other representational refinements [79].

## Endnotes

^a^Throughout this paper, we have written of our semantic annotation in terms of concepts, which refer to the classes/terms of the ontologies and terminologies being used. We are aware of published work that emphasizes that ontologies and terminologies represent reality rather than concepts [29,30], and for those readers who subscribe to this view, the word “concept” may be substituted with “class”, “universal”, or “type” throughout. Also, we note that what we call concepts are sometimes referred to in the NLP literature as “named entities”; however, since not all of the: entries in all of source ontologies are universally agreed to be “entities” (cf. processes), we again prefer the term “concept”.

^b^Among the challenges of using the BP and MF subontologies are their many cognate terms, as for many textual mentions it can be difficult to determine which is the appropriate subontology to draw from. Despite this and other challenging aspects, the annotators achieved an IAA of ~80% by the end of the project. This ambiguity is challenging for other uses of the GO as well, and we are still in contact with GO curators with regard to clarification and more consistent application of GO terms. As the semantic distinction between cognate process and function terms is practically often modest, the reported IAA is probably adequate for most uses.

^c^A small number of concepts from the terminologies were not used due to ambiguity and/or insufficiently clear definition. Details in the Methods section.

^d^The Open Biomedical Ontology consortium has agreed to use the Basic Formal Ontology (BFO) [74] as the upper-level ontology with which the domain-specific OBOs will be integrated, and in the BFO, an independent continuant, which encompasses most straightforward entities such as molecules, is essentially an entity that does not depend on another entity for its existence.

## Competing interest

The authors declare that they have no competing interests.

## Authors’ contributions

MB participated in the design of the corpus and in the creation of the annotation guidelines, selected the ontologies, participated in the hiring and training of annotators, reviewed annotator markup and addressed semantic issues, regularly met with the primary annotators, calculated interannotator agreement, and drafted the manuscript. ME participated in the design of the corpus, the creation of the annotation guidelines, and in the hiring and training of annotators and reviewed annotator markup. DE, KG, KS, and DS created the primary concept markup. WAB assembled the corpus, addressed technical issues, and outputted the articles and annotations in their various distribution formats. LEH conceived of an annotated corpus of full-length articles, provided overall direction, and contributed to the writing of the manuscript. KBC, KV, JAB, and LEH participated in the design and coordination of the project. All authors read and approved the final manuscript.

## Supplementary Material

Additional file 1 **Concept annotation guidelines for the CRAFT Corpus.** A Microsoft Word document presenting the motivations and salient attributes of the concept annotation guidelines for the CRAFT Corpus, followed by their description in detail. Click here for file

Additional file 2 **Interannotator-agreement statistics for CRAFT concept annotation projects.** A Microsoft Excel spreadsheet of the interannotator-agreement statistics for the CL, CHEBI, SO, NCBITaxon, and GO BP/CC/MF annotation projects. Click here for file

## References

[B1] Ananiadou S McNaught J Text Mining for Biology and Biomedicine 2006 Artech House, Boston, London

[B2] Hunter L Cohen KB Biomedical Language Processing: What’s Beyond PubMed? Mol Cell 2006 21 5 589 594 10.1016/j.molcel.2006.02.012 16507357 PMC1702322

[B3] Jensen LJ Šarić J Bork P Literature mining for the biologist: from information retrieval to biological discovery Nat Rev Genet 2006 7 119 129 10.1038/nrg1768 16418747

[B4] Zweigenbaum P Demner-Fushman D Yu H Cohen KB Frontiers of biomedical text mining: current progress Brief Bioinform 2007 8 5 358 375 10.1093/bib/bbm045 17977867 PMC2516302

[B5] Hersh W Information retrieval: a health and biomedical perspective 2008 3 Springer,

[B6] Bodenreider O Biomedical Ontologies in action: role in knowledge management, data integration and decision support Yearb Med Inform 2008 47 67 79 18660879 PMC2592252

[B7] Smith B Ashburner M Rosse C Bard C Bug W Ceusters W Goldberg LJ Eilbeck K Ireland A Mungall CJ Leontis N Rocca-Serra P Ruttenberg A Sansone SA Scheuermann RH Shah N Whetzel PL Lewis S The OBI Consortium The OBO Foundry: coordinated evolution of ontologies to support biomedical data integration Nature Biotech 2007 25 1251 1255 10.1038/nbt1346 PMC2814061 17989687

[B8] Curtis RK Orešič M Vidal-Puig A Pathways to the analysis of microarray data Trends Biotech 2005 23 8 429 435 10.1016/j.tibtech.2005.05.011 15950303

[B9] Khatri P Drăghici S Ontological analysis of gene expression data: current tools, limitations, and open problems Bioinform 2005 21 18 3587 3595 10.1093/bioinformatics/bti565 PMC2435250 15994189

[B10] Huang DW Sherman BT Lempicki R Bioinformatics enrichment tools: paths toward the comprehensive functional analysis of large gene lists Nucl Acids Res 2009 37 1 1 13 10.1093/nar/gkn923 19033363 PMC2615629

[B11] Leach SM Tipney H Feng W Baumgartner WA Kasliwal P Schuyler RP Williams T Spritz RA Hunter L Biomedical discovery acceleration, with applications to craniofacial development PLoS Comput Biol 2009 5 3 e1000215 10.1371/journal.pcbi.1000215 19325874 PMC2653649

[B12] Tomanek K Wermter J Hahn U A reappraisal of sentence and token splitting for life sciences documents Stud Health Technol Inform 2007 129 Pt 1 524 528 17911772

[B13] Kulick S Bies A Liberman M Mandel M McDonald R Palmer M Schein A Ungar L Winters S White P Integrated Annotation for Biomedical Information Extraction,

[B14] Coden AR Pakhomov SV Ando RK Duffy PH Chute CG Domain-specific language models and lexicons for tagging J Biomed Inform 2005 36 422 430 16337567 10.1016/j.jbi.2005.02.009

[B15] Lease M Charniak E Parsing Biomedical Literature 2005, 58 69

[B16] Roberts A Gaizauskas R Hepple M Guo Y Combining terminology resources and statistical methods for entity recognition: an evaluation 2008,

[B17] Craven M Kumlien J Constructing Biological Knowledge Bases by Extracting Information from Text Sources 1999, 10786289

[B18] Bard J Rhee SY Ashburner M An ontology for cell types Genome Biol 2005 6 2 R21 10.1186/gb-2005-6-2-r21 15693950 PMC551541

[B19] Meehan TF Masci AM Abdulla A Cowell LG Blake JA Mungall CJ Diehal AD Logical Development of the Cell Ontology BMC Bioinform 2011 12 6 10.1186/1471-2105-12-6 PMC3024222 21208450

[B20] Degtyarenko K de Matos P Ennis M Hastings J Zbinden M McNaught A Alcántara R Darsow M Guedj M Ashburner M ChEBI: a database and ontology for chemical entities of biological interest Nucl Acids Res 2008 36 Database Issue D344 D350 17932057 10.1093/nar/gkm791 PMC2238832

[B21] Sayers EW Barrett T Benson DA Bryant SH Canese K Chetvernin V Church DM DiCuccio M Edgar R Federhen S Feolo M Geer LY Helmberg W Kapustin Y Landsman D Lipman DJ Madden TL Maglott DR Miller V Mizrachi I Ostell J Pruitt KD Schuler GD Sequeira E Sherry ST Shumway M Sirotkin K Souvarov A Starchenko G Tatusova TA Wagner L Yaschenko E Ye J Database resources of the National Center for Biotechnology Information Nucl Acids Res 2009 37 Database Issue D5 15 18940862 10.1093/nar/gkn741 PMC2686545

[B22] Eilbeck K Lewis SE Mungall CJ Yandell M Stein L Durbin R Ashburner M The Sequence Ontology: a tool for the unification of genome annotations Genome Biol 2005 6 R44 10.1186/gb-2005-6-5-r44 15892872 PMC1175956

[B23] Mungall CJ Batchelor C Eilbeck K Evolution of the Sequence Ontology terms and relationships J Biomed Inform 2011 44 1 87 93 10.1016/j.jbi.2010.03.002 20226267 PMC3052763

[B24] Maglott D Ostell J Pruitt KD Tatusova T Entrez Gene: gene-centered information at NCBI Nucleic Acids Res 2011 39 Database issue D52 57 21115458 10.1093/nar/gkq1237 PMC3013746

[B25] The Gene Ontology Consortium Gene Ontology: tool for the unification of biology Nat Genet 2000 25 25 29 10.1038/75556 10802651 PMC3037419

[B26] The Gene Ontology Consortium Enhancements for 2012 Nucleic Acids Res 2010 40 Database issue D559 D564 10.1093/nar/gkr1028 PMC3245151 22102568

[B27] Verspoor K Cohen KB Lanfranchi A Warner C Johnson HL Roeder C Choi JD Funk C Malenkiy Y Baumgartner WA Ogren PV Bada M Palmer M Hunter LE A corpus of full-text journal articles is a robust evaluation tool for revealing differences in performance of biomedical natural language processing tools Accepted BMC Bioinform 2011 10.1186/1471-2105-13-207 PMC3483229 22901054

[B28] Cohen KB Lanfranchi A Corvey W Baumgartner WA Roeder C Ogren PV Palmer V Hunter L Annotation of all coreference in biomedical text: Guideline selection and adaptation 2010, 37 41

[B29] Smith B Beyond Concepts: Ontology as Reality Representation,

[B30] Smith B From concepts to clinical reality: an essay on the benchmarking of biomedical terminologies J Biomed Inform 2006 39 3 299 306 10.1016/j.jbi.2005.11.008 16293444

[B31] Bada M Hunter L Using the Gene Ontology to Annotate Biomedical Journal Articles 2009,

[B32] Bada M Hunter L Desiderata for ontologies to be used in semantic annotation of biomedical documents J Biomed Inform 2011 44 1 94 101 10.1016/j.jbi.2010.10.002 20971216

[B33] Dligach D Nielsen RD Palmer M To Annotate More Accurately or to Annotate More 2010,

[B34] Dligach D Palmer M Reducing the Need for Double Annotation 2011,

[B35] PubMed Central Open Access Articleshttp://www.ncbi.nlm.nih.gov/pmc/tools/openftlist/

[B36] GENIA Project Markup Language, http://www-tsujii.is.s.u-tokyo.ac.jp/~genia/topics/GPML/

[B37] Ogren PV Knowtator: A plug-in for creating training and evaluation data sets for Biomedical Natural Language systems 2006,

[B38] Ferrucci D Lally A Building an example application with the unstructured information management architecture IBM Systems J 2004 43 455 475

[B39] Kano Y Miwa M Cohen K Hunter L Ananiadou A Tsujii J U-Compare: a modular NLP workflow construction and evaluation system IBM J Res Dev 2011 55 3 11:1 11:10

[B40] Clark T Kinoshita J Alzforum and SWAN: The Present and Future of Scientific Web Communities Brief Bioinform 2007 8 3 163 171 10.1093/bib/bbm012 17510163

[B41] Stenetorp P Pyysalo S Topić G Ohta T Ananiadou S Tsujii J Brat: a Web-based Tool for NLP-Assisted Text Annotation,

[B42] Smith LH ETanabe L Rindflesch T Wilbur WJ MedTag: A Collection of Biomedical Annotations, 32 37

[B43] Pyysalo S Ginter F Heimonen J Björne J Boberg J Järvinen J Salakoski T BioInfer: a corpus for information extraction in the biomedical domain BMC Bioinform 2007 8 50 10.1186/1471-2105-8-50 PMC1808065 17291334

[B44] Roberts A Gaizauskas R Hepple M Demetriou G Guo Y Roberts I Setzer A Building a semantically annotated corpus of clinical texts J Biomed Inform 2009 42 950 966 10.1016/j.jbi.2008.12.013 19535011

[B45] The FetchProt Corpus Documentation and Annotation Guidelines http://fetchprot.sics.se/Corpus/ Release20051107/FetchProtCorpusDocumentationv1.0d.pdf

[B46] Fourth i2b2/VA Shared Task and Workshop, https://www.i2b2.org/NLP/Relations/

[B47] Tanabe L Xie N Thom LH Matten W Wilbur WJ GENETAG: a tagged corpus for gene/protein named entity recognition BMC Bioinform 2005 6 Suppl 1 S3 10.1186/1471-2105-6-S1-S3 PMC1869017 15960837

[B48] Kim JD Ohta T Tateisi Y Tsujii J GENIA corpus–a semantically annotated corpus for bio-textmining Bioinform 2003 19 Suppl 1 i180 i182 10.1093/bioinformatics/btg1023 12855455

[B49] Kim JD Ohta T Tsujii J Corpus annotation for mining biomedical events from literature BMC Bioinform 2008 9 10 10.1186/1471-2105-9-10 PMC2267702 18182099

[B50] Thompson P Iqbal SA McNaught J Ananiadou S Construction of an annotated corpus to support biomedical information extraction BMC Bioinform 2009 10 349 10.1186/1471-2105-10-349 PMC2774701 19852798

[B51] Alex B Grover C Haddow B Kabadjov M Klein E Matthews M Roebuck S Tobin R Wang X The ITI TXM Corpora: Tissue Expressions and Protein-Protein Interactions 2008 Proceedings of the Workshop on Building & Evaluation of Resources for Biomedical Text Mining, LREC

[B52] Smith L Rindflesch T Wilbur WJ MedPost: a part-of-speech tagger for bioMedical text Bioinform 2004 20 14 2320 2321 10.1093/bioinformatics/bth227 15073016

[B53] Yapex Collections of MEDLINE abstracts, http://www.sics.se/humle/projects/prothalt/ README_yapex_text_collection.txt

[B54] Rebholz-Schuhmann D Jimeno-Yepes AJ van Mulligen EM Kang N Kors J Milward D Corbett P Buyko E Tomanek K Beisswanger E Hahn U The CALBC Silver Standard Corpus – Harmonizing Multiple Semantic Annotations in a Large Biomedical Corpus J Bioinform Comput Biol 2010 8 1 163 179 10.1142/S0219720010004562 20183881

[B55] Pradhan S Hovy E Marcus M Palmer M Ramshaw L Weischedel R OntoNotes: A Unified Relational Semantic Representation 2007 517 526

[B56] OntoNotes Release 2.0 http://yertle.ldc.upenn.edu/Catalog/docs/LDC2008T04/OntoNotes-Release-2.0.pdf

[B57] Blaschke C Valencia A Can bibliographic pointers for known biological data be found automatically? Protein interactions as a case study Comp Funct Genom 2001 2 196 206 10.1002/cfg.91 PMC2447212 18628915

[B58] Corney DPA Buxton B Langdon WB Jones DT BioRAT: extracting biological information from full-length papers Bioinform 2004 20 17 3206 3213 10.1093/bioinformatics/bth386 15231534

[B59] Shah PK Perez-Iratxeta C Bork P Andrade MA Information extraction from full text scientific articles: Where are the keywords? BMC Bioinform 2003 4 20 10.1186/1471-2105-4-20 PMC166134 12775220

[B60] Cohen KB Johnson HL Verspoor K Roeder C Hunter LE The structural and content aspects of abstracts versus bodies of full text journal articles are different BMC Bioinform 2010 11 492 10.1186/1471-2105-11-492 PMC3098079 20920264

[B61] Bada M Livingston K Hunter L From Text to Knowledge: Toward Systematic Composition of Complex Representations 2011,

[B62] Lord PW Stevens RD Brass A Goble CA Investigating semantic similarity measures across the Gene Ontology: the relationship between sequence and annotation Bioinform 2003 19 10 1275 1283 10.1093/bioinformatics/btg153 12835272

[B63] Alterovitz G Xiang M Mohan M Ramoni MF GO PaD: the Gene Ontology Partition Database Nucleic Acids Res 2007 35 suppl 1 D322 D327 17098937 10.1093/nar/gkl799 PMC1669720

[B64] Tateisi Y Ohta T Collier N Nobata C Tsujii J Building an Annotated Corpus in the Molecular-Biology Domain 2000,

[B65] Soldatova L Liakata M An ontology methodology and CISP - the proposed Core Information about Scientific Papers JISC Project Report 2007

[B66] Cohen KB Christiansen T Hunter LE Parenthetically speaking: Classifying the contents of parentheses for text mining 2011, PMC3243264 22195078

[B67] Mouse Genome Informaticshttp://www.informatics.jax.org/

[B68] Eppig JT Blake JA Bult CJ Kadin JA Richardson JE the Mouse Genome Database Group The Mouse Genome Database (MGD): comprehensive resource for genetics and genomics of the laboratory mouse Nucl Acids Res 2012 40 Database Issue D881 D886 22075990 10.1093/nar/gkr974 PMC3245042

[B69] Smith CL Eppig JT The mammalian phenotype ontology: enabling robust annotation and comparative analysis Wiley Interdiscip Rev Syst Biol Med 2010 1 3 390 399 20052305 10.1002/wsbm.44 PMC2801442

[B70] Bada M Eckert M Palmer M Hunter LE An Overview of the CRAFT Concept Annotation Guidelines 2010 Proceedings of the Linguistic Annotation Workshop IV, Association for Computational Linguistics (ACL) Conference

[B71] Gennari JH Musen MA Fergerson RW Grosso WE Crubézy M Eriksson H Noy NF Tu SW The Evolution of Protégé: An Environment for Knowledge-Based Systems Development Internat J Human-Comp Studies 2003 58 1 89 123 10.1016/S1071-5819(02)00127-1

[B72] Sarntivijai S Ade AS Athey BD States DJ The Cell Line Ontology and its use in tagging cell line names in biomedical text AMIA Annu Symp Proc 2007 11 1103 18694200

[B73] Mani I Hu Z Jang SB Samuel K Krause M Philips J Wu CH Protein name tagging guidelines: lessons learned Comp Funct Genom 2005 6 72 76 10.1002/cfg.452 PMC2448601 18629297

[B74] Grenon P Smith B Goldberg L Pisanelli DM Biodynamic Ontology: Applying BFO in the Biomedical Domain Ontologies in Medicine 2004 Ios Press, Amsterdam 20 38

[B75] NCBI Taxonomy Databasehttp://www.ncbi.nlm.nih.gov/taxonomy

[B76] Ereshefsky M The Poverty of the Linnaean Hierarchy: A Philosophical Study of Biological Taxonomy 2001 Cambridge University Press, Cambridge

[B77] Schulz S Stenzhorn H Boeker M The ontology of biological taxa Bioinform 2008 24 13 i313 i321 10.1093/bioinformatics/btn158 PMC2718636 18586729

[B78] Bada M Eilbeck K Toward a Richer Representation of Sequence Variation in the Sequence Ontology 2010, Proceedings of the Annotation, Interpretation and Management of Mutations Workshop, 9th European Conference on Computational Biology (ECCB)

[B79] Bada M Eilbeck K Efforts toward a More Consistent and Interoperable Sequence Ontology 2012,

