# Supplementary material for: Concept annotation in the CRAFT corpus
Source: BMC Bioinformatics. 2012 Jul 9;13:161. doi: 10.1186/1471-2105-13-161 (PMC3476437; doi:10.1186/1471-2105-13-161)
Supplement: Additional file 1 — Concept annotation guidelines for the CRAFT Corpus. A Microsoft Word document presenting the motivations and salient attributes of the concept annotation guidelines for the CRAFT Corpus, followed by their description in detail. [file 1471-2105-13-161-S1.doc]

## Concept-Annotation Guidelines

Concept annotation of the CRAFT Corpus entails marking up every mention (including abbreviations and misspellings) of every *explicitly represented* concept of a given ontology/terminology such that the span(s) of text selected for each annotation are as semantically close as possible—essentially semantically equivalent—to the concept that is used to mark up the text. Thus, a mention of, *e.g.*, leukocytes is annotated with a term representing leukocytes rather than with a relatively general term, *e.g.*, a term representing cells, from a much smaller annotation schema, as is commonly done. The text that is selected for a given annotation never denotes a concept that is more specific than the concept with which it is annotated. Thus, given a terminology with a concept representing cells but without a concept representing leukocytes, a given mention of leukocytes would not be annotated, as the selected text (“leukocytes”) would be more specific than the concept with which it is annotated (*i.e.*, that representing cells). On the other hand, the concept used to annotate a textual mention may be more specific than the strict text in isolation if it is the correct context; for example, “migrates” may be marked up with GO:cell migration (GO:0016477) if this mention is within the context of cell migration, as this is the most general migration term in the GO of the correct context. We aim to identify concepts and extract assertions from biomedical text as precisely as possible, and with this annotation strategy, we can mark up concepts in text without loss of knowledge, whereas, *e.g.*, marking up “leukocytes” with a term representing cells in general would be a semantically lossy annotation. Mentions of concepts that are not explicitly represented in the terminology but are within its domain (which is often vague and subjective, as we will soon discuss) therefore are not annotated; however, this makes unambiguous which spans of text have corresponding concepts in the terminology or terminologies being used and are thereby formally captured in a semantically lossless way and which spans of text do not. In this way, it is clear which textual knowledge remains to be formally represented.

In addition to this goal of capturing textual knowledge in a semantically lossless way, this strategy makes for a more doable process of concept annotation by making it more consistent and straightforward and less subjective (though we would still not say that it is an easy process). Another strategy we considered was to annotate each textual mention denoting a concept that is subsumed by at least one concept represented in the terminology being used, as opposed to being semantically equivalent to a concept represented in the terminology. For example, given a textual mention of chromic acid and a chemical ontology that does not have a term for chromic acid but does have a term representing acids in general, this would entail annotating “chromic acid” with acid (CHEBI:37527)[[1]](#footnote-2), as this term, though not semantically equivalent to chromic acid, subsumes it. It may seem like an attractively straightforward strategy, but it is deceptively difficult to use in practice: Should an arbitrary mention of a chemical that could plausibly act as an acid or a base depending on the chemical environment (*e.g.*, bicarbonate) be annotated as an acid? Should a mention of a chemical that is typically considered an acid but could possibly act as a base (*e.g.*, sulfuric acid in the presence of a superacid) or a chemical that is typically not considered an acid but could possibly act as an acid (*e.g.*, hydroxide in the presence of a superbase) be annotated as an acid? Should a mention of a chemical that could act as an acid according to Lewis theory but not according to Brønsted-Lowry theory (*e.g.*, boron trifluoride) be annotated as an acid? It may be tempting to annotate or not annotate according to whether the chemical is acting as an acid (according to the theory to which the annotator surmises the author(s) of the paper to subscribe) in a given textual context, but this would introduce a crushing amount of work for the annotator, not to mention a significant opportunity for error; furthermore, many, if not most, mentions will not unambiguously indicate whether the chemical is acting as an acid. This is compounded by the fact that a concept may be able to be classified within more than one branch of an ontology. For example, a chemical could be classified in many different ways: organic or inorganic; neutral, charged, or radical; homoatomic or heteroatomic; small molecule or macromolecule; presence of functional groups; etc. This quickly becomes an overwhelming task, and it would be even worse for concepts that are less precisely defined than many types of chemical entities. It is much simpler and more objective to evaluate whether a given mention of a concept is semantically equivalent to a concept represented in a terminology than whether it is subsumed by one or more concepts in the terminology, and this maximizes the correctness and consistency of the semantic markup.

Another potential strategy in the case of encountering a textual mention of a concept that is not directly represented in the ontology but is thought to be within the domain of the ontology would be to annotate the mention with the root term of the ontology. This is inarguably simpler and less subjective than the aforementioned alternative strategy; however, it can still suffer from the same problems. Some ontologies have ill-defined domains, and though orthogonality is emphasized among the Open Biomedical Ontologies, it is not enforced. If an annotation project is using ChEBI to mark up chemicals, chromic acid may be easy to classify as a chemical, but what about proteins? ChEBI defines itself as a “dictionary of molecular entities focused on 'small' chemical compounds” (with the nested single quotes appearing in the original text) [1]. An annotator, even with expertise in chemistry or biochemistry, may infer from this that macromolecules such as proteins and nucleic acids are outside of the scope of ChEBI and that therefore a textual mention of a protein should not be annotated as a chemical. However, the ontology does have terms representing macromolecules, nucleic acids, and proteins, among others; there is even a small hierarchy of types of proteins. But functionally defined proteins (*e.g.*, transporters, enzymes such as kinases and transferases) are outside of the scope of ChEBI; rather, the corresponding functionalities are represented in the molecular-function subontology of the GO (*e.g.*, transporter activity, kinase activity, transferase activity). Deciding which chemicals are “of biological interest” and which are not can also be ambiguous: Atoms of all named elements are represented, including the lanthanides and actinides, most of which have little biological relevance, as are xenobiotic chemicals; indeed, any chemical could be considered xenobiotic if it found its way into in an organism. The situation becomes even more nuanced when dealing with the hierarchy of biological roles within ChEBI: Within this branch of the ontology are terms representing functionally defined small molecules, such as acids, toxins, mutagens, foods, catalysts, and antibiotics, but not, *e.g.*, antiperspirants or odorants. The point of this is not at all to denigrate ChEBI (which we consider a well-constructed ontology overall), as the broader issue of the difficulties inherent in deciding whether a given concept denoted by a mention is within the “domain” of an ontology is a general one. Rather, we are asserting that it is significantly less difficult to evaluate whether a given mention of a concept is (essentially) semantically equivalent to any concept represented in a given ontology than it is to evaluate whether it is subsumed by any concept represented in the ontology or even whether it is within the domain of the ontology: Instead of having to decide which of chromic acid, fluoroantimonic acid, protein, kinase, toxin, and odorant are within the domain of ChEBI and which are not, the annotator can more objectively note that chromic acid, proteins, and toxins are explicitly represented in ChEBI and fluoroantimonic acid, kinases, and odorants are not and mark up the text (or not) accordingly. Minimizing this difficulty affords more consistent and less subjective markup, as evidenced by our high interannotator-agreement statistics.

Any selected text span of a concept annotation must be adjacent on each of its boundaries to an appropriate delimiter. Typically, the delimiter is most often a whitespace character:

*Pygo1*: :and: :*Pygo2*: :roles: :in: :Wnt: :signaling: :in: :mammalian: :kidney: :development [PMID:17425782][[2]](#footnote-3)

Here, each colon indicates a possible boundary of a text span of an annotation. Any punctuation mark can also serve as a delimiter marking the boundary of a text span of an annotation:

Spinocerebellar ataxia 15 :(:SCA15:):,: a human autosomal dominant disorder:,: maps to the genomic region containing *ITPR1*:;: however:,: to date no causal mutations had been identified:.: [PMID:17590087]

Lastly, beginnings and ends of documents can trivially serve as boundaries of text spans of annotations. It will be shown later that a concept annotation can consist of two or more discontinuous text spans; in these cases, each boundary of each component text span must be adjacent to one of these delimiters.

It is important to note than a letter (including a non-Latin latter) or number can never serve as a delimiter. In other words, no annotation text span can begin or end between two letters, two numbers, or a number and a letter. In addition to its intuitiveness in terms of the resulting boundaries of tokens (in that adjacent letters and numbers are not split), this criterion was chosen so as to maximize the feasibility of the annotation process, as allowing splits between numbers and letters would introduce a very large amount of additional effort. In particular, every substring of every word would have to be examined for the occurrence of an ontological concept, bringing with it significant ambiguity and chance for error: Should “zyg” of “homozygous” be annotated with zygote (CL:0000365) since this morpheme denotes this concept? Should “cyto” of “cytological” be annotated with cell (GO:0005623) since this prefix denotes cells? Should “nucleo” of “nucleotide” be annotated with nucleus (GO:0005634) and/or with nucleic acid (CHEBI:33696) as this prefix could refer to either of these concepts [2]? Additionally, every letter of every abbreviation would need to be checked, further compounding the difficulty; extending our policy of annotating abbreviations (subject to our delimiter guideline), the “*I*” and “*P*” of “*ITPR1”* in Ex. 2 would be respectively annotated with inositols (CHEBI:24848) and CHEBI:phosphate group (CHEBI:32958) since these are mentioned in the gene's full name of inositol 1,4,5-triphosphate receptor 1, for example. Disallowing the splitting of tokens between numbers and letters at times prevents the annotator from creating concept markup that may be reasonable, but this is relatively rare in our experience, and this is far outweighed by its advantages of significant reduction of effort and opportunity for error. Furthermore, our span delimitation guidelines are clear and straightforward to follow by both human and computational annotators.

Each concept annotation is anchored in a single word that is the central word of the annotated concept, and we therefore call this the *anchor word*. This anchor word may be annotated by itself if it directly refers to an ontological concept. Alternately, starting from this anchor word, the annotator may consider additional spans of text to be included in the annotation, but only provided that the included text, along with the anchor word, directly refers to an ontological concept. Which additional spans of text may be considered for inclusion and which may not are strictly defined by syntax. We will present example concept annotations illustrating these syntax-based guidelines arranged by part of speech and progressively increasing in complexity. We have selected examples from each of the terminologies used in this project to illustrate that these guidelines are terminology-independent.

Since ontological concepts are lexicalized within the ontologies as nouns or noun phrases, concept annotations in natural-language text are most often—but by no means always—nouns or noun phrases.

**Unmodified Nouns.** Most straightforward are unmodified nouns that denote ontological concepts. (In all examples in this paper, annotations are underlined, and all of these are actual examples in our corpus.)

**Ex. 1:**  However, we were unable to generate DsRed1 lines, suggesting that DsRed1 is not developmentally neutral or that transgene expression cannot be sustained constitutively. [PMID:12079497]

**Ex. 2:** We have recently cloned and characterized a novel gene family named ancient conserved domain protein (ACDP) in humans. [PMID:14723793]

**Ex. 3:**  BRCA2 appears to function in recombination via interactions with the major eukaryotic recombinase RAD51. [PMID:11597317]

A noun that modifies a noun or noun phrase may be selected, as in Ex. 2, in which “transgene”, which modifies “expression” but is itself unmodified, is annotated with transgene (SO:0000902). Ex. 2, in which “humans” is annotated with Homo sapiens (NCBITaxon:9606) shows that synonyms and synonymous phrases as well as plural forms of ontological concepts are annotated. In Ex. 3, “BRCA2” is marked up with BRCA2 breast cancer 2, early onset [*Homo sapiens*] (EG:675) which shows that all appropriate abbreviations are also annotated. Note that this same Entrez Gene concept could be used to annotate a mention of hBRCA2 (*i.e.*, human BRCA2), as this species-specific concept is a direct match for this mention; however, such a mention could not be annotated with a taxon-independent BRCA2 concept (*e.g.*, the taxon-independent PR term breast cancer type 2 susceptibility protein (PR:000004804)), as hBRCA2 is a more specific concept and the “BRCA2” cannot be selected independently of the preceding “h” since there is no intervening delimiter.

**Nouns with Determiners and/or Quantifiers.** If a noun or noun phrase to be annotated has one or more determiners and/or quantifiers, they are not included in the annotation. All of the following are examples in which an unmodified noun is preceded (not immediately in some cases) by a determiner and/or simple or complex quantifier:

**Ex. 4:** The mice were 6 to 12 months old at the time of analysis and were not studied at other ages unless indicated. [PMID:11532192]

**Ex. 5:** These enzymes are expressed with a prosegment consisting of 195 residues that is cleaved off during maturation, yielding the active catalytic domains. [PMID:14609438]

**Ex. 6:** However, this latter interaction is not likely to be structurally crucial since the serine is not absolutely conserved in all CLN2-like enzymes. [PMID:14609438]

**Ex. 7:** No expression was found in fetal or adult brain, consistent with a peripheral cause of deafness. [PMID:15320950]

**Ex. 8:** In these cells its loss led to a slightly slower aggregation velocity which seems to be compensated by an increased number of platelets. [PMID:12925238]

**Ex. 9:** Thick sections were fixed in 2% (w/v) paraformaldehyde and 2% glutaraldehyde (v/v) in 0.1 M sodium cacodylate buffer pH 7.2 at RT for 24 hrs then washed several times in Tris buffered saline (0.5 M Tris, 150 mM NaCl, pH 7.4). [PMID:12546709]

**Ex. 10:** This promoter/enhancer combination has previously been shown to drive strong and widespread transgene expression in ES cells, embryos, and adult mice. [PMID:12079497]

**Ex. 11:** In contrast to wild type lenses (Figure 3L and 3Q), the alphaA/BKO lenses (Figure 3B and 3G) contained large areas devoid of cellular material, their nuclei were not limited to a well defined equatorial/bow region, and ordered radial columns of elongating fiber cells extending from the posterior capsule to the anterior epithelium were not observed. [PMID:12546709]

Ex. 4, which is marked up with Mus (NCBITaxon:10088), shows that no articles (neither definite nor indefinite) are included in concept annotations. Ex. 5, 6, 7, 8, and 9 are respectively marked up with amino-acid residues (CHEBI:33708), TPP1 tripeptidyl peptidase I [*Homo sapiens*] (EG:1200), gene expression (GO:0010467), platelet (CL:0000233), and sodium chloride (CHEBI:26710); they illustrate that no quantifiers—simple or complex, qualitative or quantitative—are included. Furthermore, neither demonstrative nor possessive determiners are included, as respectively shown in Ex. 10 and 11, which are respectively annotated with enhancer (SO:0000165) and nucleus (GO:0005634). (In Ex. 6 and 10, the determiner or quantifier actually modifies a larger noun phrase than the single annotated noun, but we include such examples here for completeness.)

**Nouns with Adjectives and/or Adjectival Phrases.** If the anchor noun has one or more adjectives, an adjective is included in the annotation only if it is needed to annotate the text span with a direct concept in the ontology:

**Ex. 12:** All 5' primers contain a Kozak consensus translation initiation site upstream of the ATG for increased translation efficiency in eukaryotic cells. [PMID:12079497]

**Ex. 13:** Characterization of the *Mcoln1* alternative splice variant [PMID:11897010]

**Ex. 14:** At the cellular level annexin A7 can be detected in the cytosol, at the plasma membrane, around the nucleus, at vesicular structures including adrenal chromaffin granules, and at the t-tubule system. [PMID:14675480]

**Ex. 15:** Dopamine D2 receptor-mediated signaling contributes to the acquisition of odor discrimination/associative learning. [PMID:15061865]

**Ex. 16:** The *Acdp3* and *Acdp4* genes map to chromosome 1 within one BAC clone (RP23-294I17), proximal to marker *D1Mit171* (17.4 cR). [PMID:14723793]

In Ex. 12, the adjective “eukaryotic” is included in the annotation since its inclusion results in a semantic match to eukaryotic cell (CL:0000255). In Ex. 13, both “alternative” and “splice” (along with intervening spaces) are included since their inclusion results in a match to alternatively_spliced_transcript (SO:1001187), but in Ex. 14, “chromaffin” but not “adrenal” is included since the inclusion of the former results in a match to the CC term chromaffin granule (GO:0042583) and there is no more specific concept in the GO representing adrenal chromaffin granules. Expanding on this, in Ex. 15, “Dopamine” and “receptor-mediated” but not “D2” are included since the inclusion of the first two results in a match to the BP term dopamine receptor signaling pathway (GO:0007212) and there is no more specific concept in the GO for the dopamine D2 receptor signaling pathway; this is an example of a *discontinuous annotation*, in which one annotation is composed of two or more noncontiguous text spans. (This example also illustrates that adjectival phrases such as “receptor-mediated” are evaluated for inclusion like simple adjectives.) Adjectives and/or adjectival phrases can be included with independently evaluated conjuncts of coordinated phrases provided this leads to a match; the adjective or adjectival phrase may immediately precede the conjunct or may be discontinuous, as in Ex. 16, which is annotated with Cnnm3 cyclin M3 [*Mus musculus*] (EG:94218).

**Nouns with Prepositional Phrases.** In addition to adjectives and adjectival phrases, a prepositional phrase is included in annotations if its inclusion results in a match to an ontological concept. If a prepositional phrase is included, the preposition and the head noun of the object of the phrase must be included; other constructs modifying this head noun are recursively included if their inclusion results in a match:

**Ex. 17:** ADAMs are also involved in cell-cell or cell-matrix adhesion through their interaction with integrins or syndecans. [PMID:15873656]

**Ex. 18:** We have demonstrated a translocation of Annexin A7 to nuclei of cells in early murine brain development and the presence of Annexin A7 in nuclei of neuronal cells in the adult animal. [PMID:15819996]

**Ex. 19:** Red fluorescence was often aggregated in what appeared to be perinuclear regions of the cytoplasm. [PMID:12079497]

**Ex. 20:** Prior to initiation of DNA replication, Mcm helicase may adopt a double hexameric complex at the partially melted origin region, and may catalyze concurrent unwinding of duplex DNA into both directions, while stably associated with the origins of DNA replication. [PMID:15917436]

**Ex. 21:** During *Drosophila* neuroblast generation, the transcription factor Hunchback controls specification and differentiation of early-born neuroblasts. [PMID:15836427]

Ex. 17, which is annotated with the MF term integrin binding (GO:0005178), shows a basic case in which an adjacent prepositional phrase simply consists of the preposition and an unmodified object. A prepositional phrase not adjacent to the noun it modifies may be selected to form a discontinuous annotation, as in Ex. 18, which is marked up with the BP term nuclear import (GO:0051170). Modifiers of the object noun of a selected prepositional phrase are recursively evaluated for inclusion: Ex. 19, which is annotated with the CC term perinuclear region of cytoplasm (GO:0048471), illustrates that a determiner of the object noun is recursively not included (nor are any quantifiers). In Ex. 20, the object noun is premodified with “DNA”, which is included in the annotation since the entire phrase “origins of DNA replication” is synonymous with origin_of_replication (SO:0000296), but in Ex. 21, the premodifying “early-born” is not included in the annotation of the BP term neuroblast fate specification (GO:0014018) since there is no more specific ontological concept that captures this knowledge. Ex. 21 also illustrates that the conjuncts of coordinated phrases may be evaluated independently with prepositional phrases; here, “of early-born neuroblasts” modifies both “specification” and “differentiation”, the former of which is selected along with the preposition and object of the prepositional phrase, creating a discontinuous annotation.

**Nouns with Relative Clauses.** Neither restrictive nor nonrestrictive relative clauses are included in annotations, even if the annotator believes inclusion would result in a match to an ontological concept. (In practice, we have found it rare for this to happen, as inclusion of a given relative clause typically results in a concept that is much more specific than any concept of a given ontology.)

**Ex. 22:** The model presented here indicates a very open and accessible active site that is almost completely conserved among all known CLN2 enzymes. [PMID:14609438]

**Ex. 23:** By positional cloning and gene targeting, we identify an allele of *droopy ear*, *de^H^*, as a loss of function for *Tbx15*, which encodes a T-box transcription factor expressed in a dynamic and spatially restricted manner in the developing skin and musculoskeletal system. [PMID:14737183]

**Ex. 24:** The second paper providing evidence, by Davies et al, is a biochemical study of the interaction between the homologous recombinase RAD51 and a peptide consisting of one of the eight BRC repeats from human BRCA2. [PMID:11597317]

Ex. 22, 23, and 24 are annotated with polypeptide_catalytic_motif (SO:0100019), Tbx15 T-box15 [*Mus musculus*] (EG:21384), and peptides (CHEBI:16670), respectively.

**Nouns in Apposition.** A noun or noun phrase in either restrictive or nonrestrictive apposition with one or more other nouns and/or noun phrases may often be thought of as modifying the other(s). However, apposition is sufficiently unique and common in biomedical writing that we have addressed it separately. Nouns and noun phrases in restrictive apposition are evaluated for annotation independently of the constructs with which they are in apposition, and no annotation should encompass more than one of these appositive constructs; they are otherwise evaluated just as other nouns and noun phrases.

**Ex. 25:** The model of human CLN2 was built using the structure of sedolisin complexed with the inhibitor pseudo-tyrostatin. [PMID:14609438]

**Ex. 26:** In this study, we find that the DM domain protein DMRT7 is required for male germ cells to complete meiotic prophase but is dispensable in the female germ line. [PMID:17447844]

**Ex. 27:** The most efficiently processed peptide consisted of 14 amino acids, with the tripeptide Asp-Arg-Val removed from its N terminus. [PMID:14609438]

For example, in Ex. 25, the first appositive construct, “the inhibitor”, has been evaluated independently of the second appositive construct, “pseudo-tyrostatin”. Any or all of the independently evaluated appositive constructs may be annotated, dependent on a match to an ontological concept. Note that a part of an appositive construct can be annotated, as in Ex. 27. Ex. 25, 26, and 27 are respectively annotated with inhibitor (CHEBI:35222), Dmrtc2 doublesex and mab-3 related transcription factor like family C2 [*Mus musculus*] (EG:71241), and aspartic_acid (SO:0001453).

Nouns and noun phrases in nonrestrictive apposition are also evaluated independently. In such cases where the appositive elements are alternate names for the same ontological concept, separate annotations are made for each of these.

**Ex. 28:** Here, we generated mice deficient for *Trip13*, the ortholog of *PCH2*, and evaluated whether it also plays a role in the pachytene checkpoint. [PMID:17696610]

In Ex. 28, “*Trip13*” is annotated with Trip13 thyroid hormone receptor interactor 13 [*Mus musculus*] (EG:69716) and is in nonrestrictive apposition with “the ortholog of *PCH2*”; however, the latter is not similarly annotated since it is descriptive rather than definitive. An especially prominent use of nonrestrictive apposition in biomedical articles is the juxtaposition of alternate names and/or abbreviations of a given entity, particularly genes and gene products and other types of chemicals. In Ex. 29a/b, the sentence has been repeated for each annotation to emphasize that these are two separate annotations of ethyl nitrosourea (CHEBI:23995) rather than a single discontinuous annotation.

**Ex. 29a:** We have identified ethyl-nitrosourea (ENU)-induced point mutations in three alleles of *mon*, each of which generates a premature stop codon. [PMID:15314655]

**Ex. 29b:** We have identified ethyl-nitrosourea (ENU)-induced point mutations in three alleles of *mon*, each of which generates a premature stop codon. [PMID:15314655]

Appositives were also annotated on a separate layer as part of the coreference resolution project. This is discussed elsewhere.

**Verbs.** If an ontological concept represents a process, any verbal form for the process is annotated with the concept. Verbs are typically annotated by themselves, though a given adverb is included if its inclusion results in a match to a concept.

**Ex. 30:** This PCR assay produces a 392 bp product from the *de^H^* chromosome and a 595 bp product from the nonmutant chromosome. [PMID:14737183]

**Ex. 31:** When E-cadherin is transcriptionally down-regulated, associated adhesion proteins with dual functions in signaling are released from cell-cell contacts, a process which we demonstrate leads to Ras-MAPK activation. [PMID:15630473]

Ex. 30 and 31 are respectively annotated with the BP terms biosynthetic process (GO:0009058) and negative regulation of transcription (GO:0016481).

**Adjectives.** For an ontological concept lexicalized as a (typically singular) noun or noun phrase, its adjectival form or a semantic equivalent is annotated with the concept, as in Ex. 32, which is annotated with Mammalia (NCBITaxon:40674).

**Ex. 32:** It is not known if these abnormalities affect mammalian IOP. [PMID:11532192]

Some ontologies, such as the SO, do have terms, usually representing qualities, that are lexicalized as adjectives or adjectival phrases; such terms are used to directly annotate an adjective or adjectival phrase in the text, as in Ex. 33, which is annotated with conserved (SO:0000856).

**Ex. 33:** A model of tripeptidyl-peptidase I (CLN2), a ubiquitous and highly conserved member of the sedolisin family of serine-carboxyl peptidases [PMID:14609438]

**Adverbs.** Corresponding adverbial forms of ontological concepts are annotated, although this is rare. In Ex. 34, “transcriptionally” is annotated with the BP term transcription (GO:0006350).

**Ex. 34:** When E-cadherin is transcriptionally down-regulated, associated adhesion proteins with dual functions in signaling are released from cell-cell contacts, a process which we demonstrate leads to Ras-MAPK activation. [PMID:15630473]

**Overlapping and nesting.** The selected text of an annotation *overlaps* the selected text of another annotation if the two annotations share some text. *Nesting* is a type of overlapping in which the selected text of an annotation is a proper subset of the selected text of another annotation, *i.e.*, all of the text of the former is included in the latter. The terms of the ontologies dictate whether overlapping or nesting annotations are created.

The central rule for nesting of annotations is that a nested annotation is created only if it is to be annotated with a term that is *not* a superclass of the nesting annotation. This is trivial if the terms for nesting and nested annotations are from different ontologies, as one cannot be a superclass of the other (assuming the ontologies have not been merged), as Ex. 35a and 35b, which are respectively annotated with the BP term myeloid cell differentiation (GO:0030099) and myeloid cell (CL:0000763). However, there is not a separate annotation of the nested “differentiation” with the BP term cell differentiation (GO:0030154), since this is a superclass of myeloid cell differentiation (GO:0030099).

**Ex. 35a:** In *mon* homozygous mutants, there is a severe block in maturation at the proerythroblast stage, whereas differentiation of myeloid cells is normal. [PMID:15314655]

**Ex. 35b:** In *mon* homozygous mutants, there is a severe block in maturation at the proerythroblast stage, whereas differentiation of myeloid cells is normal. [PMID:15314655]

There are no corresponding restrictions for overlapping annotations that are not nesting/nested annotations, and all such overlapping occurrences are appropriately annotated. For example, in Ex. 36a/b, which are respectively annotated with the MF terms calcium ion binding (GO:0005509) and phospholipid binding (GO:0005543), the text spans for the two annotations overlap (sharing “binding”), but neither is nested within the other.

**Ex. 36a:** Annexin A7 is a Ca^2+^- and phospholipid-binding protein expressed as a 47 and 51 kDa isoform, which is thought to be involved in membrane fusion processes. [PMID:12925238]

**Ex. 36b:** Annexin A7 is a Ca^2+^- and phospholipid-binding protein expressed as a 47 and 51 kDa isoform, which is thought to be involved in membrane fusion processes. [PMID:12925238]

**Multiply Annotated Text**. If the annotator believes that more than one term may apply to a given text span, an annotation is created for each of these terms, provided that neither term is a superclass of the other, as above. Multiple annotation may result from ambiguity, as in Ex. 37, in which two annotations for “insertion” are created, one using insert (SO:0000046) (representing the process of insertion) and the other using insertion (SO:0000667) (representing the inserted sequence). Alternately, multiple annotation may result from the fact that the same concept is represented in multiple ontologies, as in Ex. 38, in which two annotations for “mRNAs” are created, one using mRNA (SO:0000234) and the other using messenger RNA (CHEBI:33699).

**Ex. 37:** The insertion caused an exon 3 duplication between exons 5 and 6 that creates a coding region frameshift resulting in a premature termination at amino acid 255. [PMID:15760270]

**Ex. 38:** Are some olfactory receptor mRNAs more stable than others, leading to higher transcript levels per expressing cell? [PMID:14611657]

# References

1. Degtyarenko K, de Matos P, Ennis M, Hastings J, Zbinden M, McNaught A, Alcántara R, Darsow M, Guedj M, Ashburner M: **ChEBI: a database and ontology for chemical entities of biological interest.**  *Nucl Acids Res* 2008, **36** Database Issue:D344-D350.
2. **Oxford English Dictionary, nucleo-** [http://www.oed.com/view/Entry/128944#eid34381099]

1. Throughout this document, each terminological concept is rendered in fixed-width type and is parenthetically followed by its namespace and ID. [↑](#footnote-ref-2)
2. For each example, the PubMed ID of the biomedical article from which it is extracted is indicated. [↑](#footnote-ref-3)
